# SCORE Operational Research on Moving toward Interruption of Schistosomiasis Transmission

**DOI:** 10.4269/ajtmh.19-0825

**Published:** 2020-05-12

**Authors:** Carl H. Campbell, Sue Binder, Charles H. King, Stefanie Knopp, David Rollinson, Bobbie Person, Bonnie Webster, Fiona Allan, Jürg Utzinger, Shaali M. Ame, Said M. Ali, Fatma Kabole, Eliézer K. N’Goran, Fabrizio Tediosi, Paola Salari, Mamadou Ouattara, Nana R. Diakité, Jan Hattendorf, Tamara S. Andros, Nupur Kittur, Daniel G. Colley

**Affiliations:** 1 Schistosomiasis Consortium for Operational Research and Evaluation, Center for Tropical and Emerging Global Diseases, University of Georgia, Athens, Georgia;; 2 Center for Global Health and Diseases, Case Western Reserve University, Cleveland, Ohio;; 3 Swiss Tropical and Public Health Institute, Basel, Switzerland;; 4 University of Basel, Basel, Switzerland;; 5 Department of Life Sciences, Wolfson Wellcome Biomedical Laboratories, Natural History Museum, London, United Kingdom;; 6 London Centre for Neglected Tropical Disease Research, Imperial College Faculty of Medicine, London, United Kingdom;; 7 Public Health Laboratory - Ivo de Carneri, Pemba, United Republic of Tanzania;; 8 Neglected Tropical Diseases Unit, Ministry of Health Zanzibar, Unguja, United Republic of Tanzania;; 9 Centre Suisse de Recherches Scientifiques en Côte d’Ivoire, Abidjan, Côte d’Ivoire;; 10 Unité de Formation et de Recherche Biosciences, Université Félix Houphouët-Boigny, Abidjan, Côte d’Ivoire;; 11 Department of Microbiology, University of Georgia, Athens, Georgia

## Abstract

As part of its diverse portfolio, the Schistosomiasis Consortium for Operational Research and Evaluation (SCORE) included two cluster-randomized trials evaluating interventions that could potentially lead to interruption of schistosomiasis transmission (elimination) in areas of Africa with low prevalence and intensity of infection. These studies, conducted in Zanzibar and Côte d’Ivoire, demonstrated that multiyear mass drug administration (MDA) with praziquantel failed to interrupt the transmission of urogenital schistosomiasis, even when provided biannually and/or supplemented by small-scale implementation of additional interventions. Other SCORE activities related to elimination included a feasibility and acceptability assessment of test–treat–track–test–treat (T5) strategies and mathematical modeling. Future evaluations of interventions to eliminate schistosomiasis should recognize the difficulties inherent in conducting randomized controlled trials on elimination and in measuring small changes where baseline prevalence is low. Highly sensitive and specific diagnostic tests for use in very low–prevalence areas for schistosomiasis are not routinely available, which complicates accurate measurement of infection rates and assessment of changes resulting from interventions in these settings. Although not encountered in these two studies, as prevalence and intensity decrease, political and community commitment to population-wide MDA may decrease. Because of this potential problem, SCORE developed and funded the T5 strategy implemented in Egypt, Kenya, and Tanzania. It is likely that focal MDA campaigns, along with more targeted approaches, including a T5 strategy and snail control, will need to be supplemented with the provision of clean water and sanitation and behavior change communications to achieve interruption of schistosome transmission.

## INTRODUCTION AND OVERVIEW OF SCORE PORTFOLIO IN ELIMINATION

The Schistosomiasis Consortium for Operational Research and Evaluation (SCORE) was started in late 2008 with a grant from the Bill & Melinda Gates Foundation (BMGF).^1^ The SCORE portfolio contains several efforts designed to increase knowledge about how to move from morbidity control toward elimination of schistosomiasis. The initial grant included funding for a field study comparing approaches to elimination, which became the Zanzibar elimination study, described in the following section. The grant also included support to develop and test better tools for mapping of *Schistosoma mansoni* infections and for a test with high sensitivity and specificity that would allow for diagnosis of schistosomiasis in an individual with a high degree of confidence. These tools are needed to move toward interruption of schistosomiasis transmission (elimination). SCORE’s work on developing and evaluating mapping and diagnostic tools is described in other articles in this supplement.^2,3^

In 2012, the World Health Assembly (WHA) issued resolution WHA 65.21, which called for the expansion of schistosomiasis control programs and to “initiate elimination campaigns where appropriate.”^4^ Following this, in 2013, SCORE received supplemental funding from the BMGF to build on data and experiences obtained by SCORE thus far and expand operational research on elimination of schistosomiasis. In addition to results of the SCORE elimination study on Zanzibar funded with the original grant, two of the studies supported by the supplemental funding are briefly described in this article—a multiyear field study on interruption of seasonal transmission of *Schistosoma haematobium* in Côte d’Ivoire and feasibility assessments of a test–treat–track–test–treat (T5) strategy in Egypt, Kenya, and Tanzania.

The main objective of this study was to provide an overview and summary of the SCORE-funded efforts pertaining to moving toward elimination (interruption of schistosomiasis transmission). These include the following:

1. the Zanzibar elimination study;
2. the *S. haematobium* seasonal transmission elimination study in Côte d’Ivoire;
3. the T5 strategy; and
4. modeling using SCORE data.

Additional intervention studies were planned for Burundi and Rwanda, where only *S. mansoni* is endemic. Unfortunately, after countrywide mapping was completed in each country,^2,3^ the studies could not be implemented because of the lack of high-level support in Rwanda and political instability in Burundi. However, the mapping surveys yielded invaluable data about the performance of both the point-of-care circulating cathodic antigen (POC-CCA) urine cassette test and the laboratory-based up-converting phosphor-lateral flow circulating anodic antigen (UCP-LF CAA) assay in areas of low prevalence.^5–7^

### The Zanzibar elimination study.

#### Background

In July 2009, the SCORE secretariat convened a meeting to inform the design of the SCORE schistosomiasis elimination study and brainstorm about where such a study might be conducted. The discussions covered both elimination of transmission (zero new infections) and elimination as a public health problem, as defined by the World Health Organization (WHO).^8^

Participants developed the criteria for potential study sites, including a starting prevalence low enough that elimination could be considered a next step, but not so low that the study would focus on issues around verification of the interruption of transmission; adequate population size; recent experience with mass drug administration (MDA); capacity to conduct research; and high-level political will. Interventions discussed included MDA, implementation of water and sanitation improvement, snail control, and education and behavioral change efforts.

After this meeting, to find the optimal study site, SCORE reviewed documents, held discussions with neglected tropical disease (NTD) control leaders, and visited Botswana and Uganda. Candidate countries were removed from consideration for several reasons, such as the lack of political commitment in Botswana, and concerns about relevance or acceptability in Africa of results were the study to be conducted in the Americas.

Zanzibar was a strong candidate for the study because of documented low *S. haematobium* prevalence following many years of MDA, political commitment reaching to the level of the president, and an easily definable geographic area with clear boundaries. *Schistosoma haematobium* prevalence by the standard urine filtration method was documented among school-aged children on Unguja and Pemba islands, the main islands of Zanzibar, at 8% and 15%, respectively, in a 24-school survey conducted in 2011.^9^ In addition, detailed information was available about the intermediate host snail species *Bulinus globosus*, responsible for transmission on the islands.^10,11^ However, there were concerns about whether results from research on islands would be generalizable to the African mainland. These were allayed in discussions with the WHO and key individuals involved in elimination and control efforts.^1^

In December 2010, representatives from the WHO, the Zanzibar NTD Control Program, other Zanzibar government groups, the Public Health Laboratory-Ivo de Carneri on Pemba, the SCORE secretariat, and other SCORE members (e.g., the Natural History Museum [NHM] and the Schistosomiasis Control Initiative [SCI] at Imperial College) met on Zanzibar to review existing data and develop the specific operational research plan. This meeting was followed by a meeting with government leadership, including the president of Zanzibar, all of whom expressed their commitment to this elimination effort.

At the same time, a broad alliance was formed with a goal of eliminating urogenital schistosomiasis in Zanzibar. This alliance became known as the “Zanzibar Elimination of Schistosomiasis Transmission” (ZEST) alliance.^12,13^

The Zanzibar government’s National Plan for Schistosomiasis, which was endorsed by SCORE and ZEST, included biannual praziquantel MDA as the primary intervention. It was decided that the SCORE study would be a cluster-randomized trial conducted within the ZEST and within the context of the National Plan for Schistosomiasis.

#### Methods and interventions

The Zanzibar elimination study compared three interventions: biannual MDA alone, biannual MDA + snail control, and biannual MDA + behavior change interventions. Each study arm included 15 shehias on Unguja and 15 on Pemba, for a total of 90 shehias.^9^ Interventions were conducted for 5 years, starting in 2012 and ending in 2017.^14^

The main study outcomes were prevalence and intensity at Year 6 (2017) among 9- to 12-year-old children.^15^ Annually, efforts were made to survey 100 children aged 9–12 years in each of the 90 study schools with a single urine filtration for the presence and number of *S. haematobium* eggs. The statistical analysis plan for Zanzibar is included as a Supplemental file (S1). Additional results available in other publications include urine prevalence and intensity among adults, first-year students at the baseline and at the end point, and schistosome population genetics.^14–16^

#### Mass drug administration

Biannual MDA was conducted by the NTD Program of the Ministry of Health Zanzibar, according to the National Plan.^9^ Several issues were encountered related to the implementation of MDA. These included delays in program funding and difficulty coordinating MDAs with school schedules and local events.^17,18^ Nevertheless, coverage among students in study schools on Pemba was above 75% in all years. On Unguja, coverage was below 75% in years 1 and 2, but reached 97% in year 3.^16,19^

#### Snail control

From 2012 to 2017, human–water contact sites (HWCS) in shehias in the snail control arm were surveyed up to five times a year for the presence of *Bulinus* snails.^16^ Over the study period, 167 HWCS were identified on Pemba and 121 HWCS on Unguja.^20^ *Bulinus* snails were found at least once in 60 HWCS on Pemba and 71 HWCS on Unguja. On Pemba, the number of identified HWCS remained constant over the study period, and over the entire study period, 80% of sites where *Bulinus* snails were found were treated.^15^ On Unguja, the number of HWCS identified increased considerably over the study period primarily because of heavy rains and greater community assistance. After an initial start-up period, niclosamide treatment coverage of HWCS with snails was consistently > 70%. There was no clear trend of a decline in the number of snails, despite the application of molluscicide. Only nine of 5,578 (0.2%) snails collected during the study years on Pemba and 35 of 4,007 (0.9%) snails on Unguja were found to shed *Schistosoma* cercariae.

Snail control involved focal application of niclosamide in water contact sites only where snails were detected. This reduces ecologic disruption, but it also means that infected snails may remain or can return from untreated sites to contaminate water contact sites. Because *Bulinus* are hermaphroditic, a single snail surviving mollusciciding can quickly repopulate and, hence, re-infest the treated area.^15^ As indicated in Allan et al.,^20^ on Zanzibar, snails often returned quickly after mollusciciding. Another issue was the large number of natural open freshwater bodies and their remote locations, often far from roads, so that heavy equipment had to be moved by foot into a remote environment that was difficult to access.

#### Behavioral interventions

The approach selected as the behavior change intervention process—human-centered design—engages community members from the earliest stages of design,^21–23^ which increases participation and acceptance and helps ensure the interventions are culturally appropriate and match the perceived needs of the community. A disadvantage is that the design phase, although an inherent part of the intervention, took a long time in the Zanzibar study, so these interventions were not implemented until well into the study. Furthermore, not all components were implemented as extensively as desired. For example, no washing platforms were installed until the third year (2014–2015) of the study, and even then, they were not installed in all the shehias in the behavioral intervention arm.

Community participants in the design phase focused on three interventions as potentially useful for preventing *S. haematobium* transmission: (i) classroom-based interventions that would engage the whole school using interactive teaching methods (new to most in Zanzibar); (ii) school-wide outdoor interventions focused on safe play, which included teaching about schistosomiasis and its prevention as part of games; and (iii) community-based structural interventions, such as designing and building community urinals to provide an alternative to urinating in water and laundry-washing platforms to reduce exposure to potentially contaminated water.^23,24^

A cross-sectional evaluation toward the end of the study assessed knowledge and self-reported behaviors in 1,451 children from four of the 30 behavioral intervention schools and four schools from arms that had not received these interventions. Children from the intervention schools had considerably greater knowledge about *S. haematobium* transmission and prevention than children from the comparison schools. For example, they significantly more often identified key knowledge indicators such as washing clothes in the river as risk for disease transmission or the “blood fluke” as the parasite responsible for illness. On the other hand, children in comparison schools more often “incorrectly” identified the snail as the parasite that causes illness inside the body. Both groups of children displayed equally high levels of knowledge of praziquantel for treatment of disease. In terms of behavior change, significantly more children from intervention schools reported that they now take praziquantel when they had not before and that they had stopped washing and bathing in the river.

A product developed as part of the SCORE behavioral research was an intervention tool kit created with help from teachers and community members on Zanzibar. It is now available in both English and Kiswahili. It includes (i) interactive lesson plans for classroom use; (ii) an activities packet that includes games, and performances that engage students in active learning about schistosomiasis; (iii) a handout for parents entitled *Schistosomiasis and Your Child*; (iv) a flip chart that teachers can use to guide discussions about schistosomiasis; (v) slide sets that can be used to train teachers on active learning and on the use of the urogenital schistosomiasis tool kit; and (vi) materials and guidance documents necessary to conduct teacher training even when there is no electricity for slide presentations. The English version of this tool kit is available at http://TinyUrl.com/SchistoToolkit.

#### Parasitologic results

From the baseline survey in 2012 to 2017, the overall *S. haematobium* prevalence decreased from 8.2% to 1.7% on Pemba and from 4.1% to 1.7% on Unguja in 9- to 12-year-old children. In 2017, heavy infection intensities (≥ 50 eggs of *S. haematobium* per 10 mL of urine) were found in only 0.4% and 0.3% of 9- to 12-year-old children from Pemba and Unguja, respectively.

**Figure 1.**
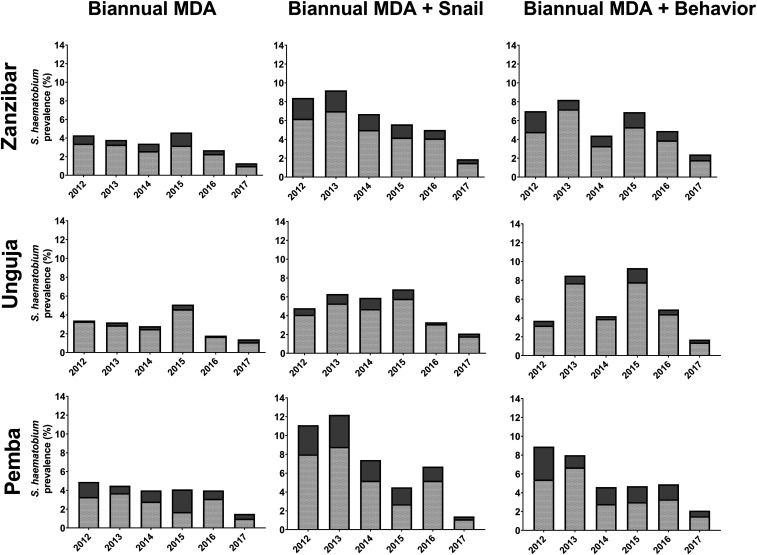
Annual change in *Schistosoma haematobium* prevalence and infection intensity in 9- to 12-year-old children, stratified by study arm, overall on Zanzibar and by island (Unguja and Pemba). Bar charts present the prevalence: dark gray solid parts present the percentage with heavy infection intensity (≥ 50 eggs per 10 mL of urine).

Figure 1 shows the annual change in *S. haematobium* prevalence and infection intensity in Zanzibar, stratified by intervention arm and island among 9- to 12-year-old children. On Pemba, the baseline prevalence in the biannual MDA-only, biannual MDA + snail control, and biannual MDA + behavior change arms was 4.9%, 11.1%, and 8.9%, respectively. These prevalence levels decreased to 1.5%, 1.4%, and 2.1%, respectively, in 2017. On Unguja, the baseline prevalence in the biannual MDA-only, biannual MDA + snail control, and biannual MDA + behavior change arms was 3.4%, 4.8%, and 3.7%, respectively. These levels decreased to 1.4%, 2.1%, and 1.7%, respectively, in 2017.

From 2015 until 2017, there was a trend of reduced odds of *S. haematobium* infection among 9- to 12-year-old children attending schools in the shehias receiving snail control.^16^ However, this difference was not statistically significant when the snail control arm was compared with the other intervention arms. There was no obvious impact of behavioral interventions on the *S. haematobium* prevalence and intensity in children.^15,16^

The study also demonstrated spatial and temporal heterogeneity in *S. haematobium* prevalence and, hence, transmission. Whereas some schools had low numbers of infected children in all years and many of those schools had zero prevalence at the end of the study in 2017, there were other schools where the prevalence decreased in some years but then rose to higher levels in other years.^16^ Of note, in 2017, there were six schools on Unguja and three on Pemba that had a prevalence > 5% in 9- to 12-year-old children, but only one school had a prevalence > 10%.

In an additional analysis, we explored the time taken for shehias on Pemba and on Unguja to reach a zero level of *S. haematobium* prevalence among 9- to 12-year-old children. There were 35 year-to-year conversion events from infected to “zero-level” prevalence in the shehias on Pemba that had at least one child with schistosomiasis in a given year and 36 events in the study shehias on Unguja.

**Figure 2.**
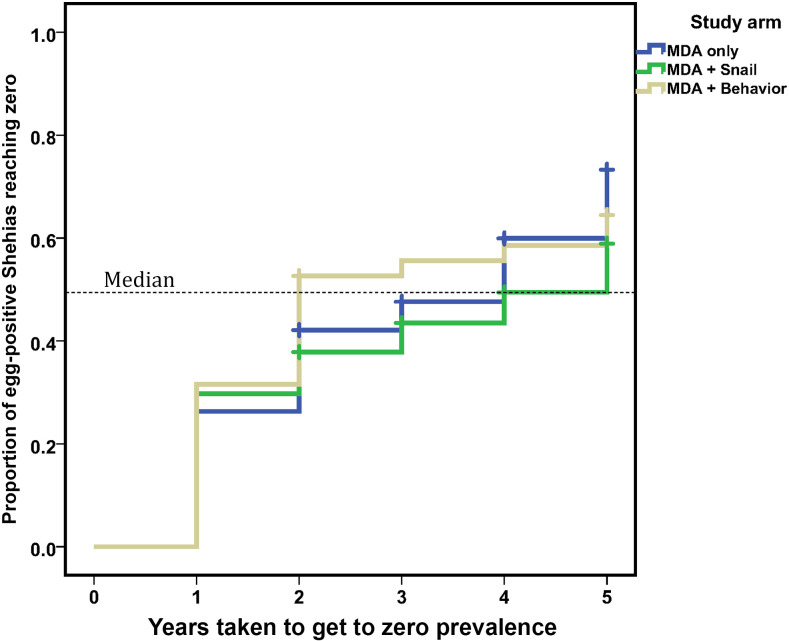
Kaplan–Meier plot showing the time needed for *Schistosoma haematobium*–endemic shehias on Zanzibar to reach a zero-prevalence status among 9- to 12-year-old children, stratified by intervention arm. Shown separately are the rates for communities given biannual mass drug administration (MDA)–only (26 events in 38 eligible schools), biannual MDA + snail control (21 events in 37 eligible schools), and biannual MDA + behavior change interventions (24 events in 38 eligible schools).

The median time taken by a shehia with infected 9- to 12-year-old children to reach a zero-prevalence status was not significantly different between arms (4 years on Pemba versus 3 years on Unguja). Although the median time for an infected shehia to reach zero was 2 years in the biannual MDA + behavior change arm, 4 years in the biannual MDA-only arm, and 5 years in the biannual MDA + snail control arm; these differences were not statistically significant. By arm, 32%, 43%, and 37% of shehias with infected 9- to 12-year-old children in the biannual MDA-only, biannual MDA + snail control, and biannual MDA + behavior change arms, respectively, failed to reach zero prevalence at any time during the period of the study. A graphic plot of the time taken to get to zero prevalence, stratified by intervention arm, is shown in Figure 2.

#### Cost evaluation

A recent SCORE-funded literature review of studies that have estimated costs of schistosomiasis interventions showed the paucity of reliable estimates.^25^ SCORE attempted an evaluation of the costs of the different interventions in the Zanzibar elimination study, the parasitologic survey activities, and for managing the programmatic activities. An effort was made to exclude costs related to research. Data were obtained from the accounting information system of the SCORE ZEST partners after fieldwork was completed. Unfortunately, the way the administrative data were collected and stored was not useful for our purpose, emphasizing the need for establishing intervention cost data systems that operate at least partially independently or in complementary ways to administrative ones.

### The *S. haematobium* seasonal transmission elimination study.

#### Background

In 2015, SCORE funded a team at Université Félix Houphouët-Boigny (Abidjan, Côte d’Ivoire), working in partnership with a team at the Swiss Tropical and Public Health Institute (Basel, Switzerland), to conduct a study on eliminating urogenital schistosomiasis in an area with seasonal transmission in Côte d’Ivoire. After an extensive eligibility survey over a wide segment of Côte d’Ivoire, the study enrolled 64 villages in the northern and central parts of the country that met the final study criteria of 4% or greater prevalence among 13- to 14-year-old schoolchildren.

The Schistosomiasis Control Initiative (SCI) and the national schistosomiasis control program agreed to jointly implement MDA, and the NHM agreed to provide expertise on snail surveys and mollusciciding.^26^

#### Methods and interventions

The study consisted of 3 years of intervention using a cluster-randomized design and had four arms, with 16 villages per arm:

1. annual MDA with praziquantel before the peak transmission season (October/November);
2. annual MDA with praziquantel after the peak transmission season (March/April);
3. biannual MDA, once before and once after peak transmission (October/November and March/April); and
4. annual MDA before peak transmission (October/November) coupled with chemical snail control using niclosamide (three applications per year; before, during, and shortly after peak transmission).

Annually, efforts were made to collect urine and stool samples in each study village from 100 children aged 9–12 years, 50 adults aged 20–55 years, and 50 first-grade children aged 5–8 years. Snails were collected from villages in arm 4 before, during, and after the peak transmission period.^27^ The primary outcome of interest was the change in prevalence and intensity over time among children aged 9–12 years.

#### Mass drug administration

Attempts were made to treat as much of the population older than 5 years with praziquantel as possible. Trained teachers treated students at school, whereas adults and out-of-school children were treated at home by community health workers.

#### Snail control

This study represents the first time that snail control using niclosamide was implemented in Côte d’Ivoire. Hence, considerable efforts were required before the study’s start to gain high-level government approval for the use of niclosamide, train teams to conduct mollusciciding, and obtain local community support for the snail intervention.

Details of the snail evaluations and niclosamide treatments are in another article in this supplement.^20^ A total of 164 HWCS were identified. The highest number of HWCS was found at the end of the rainy season. As in Zanzibar, mollusciciding with niclosamide was only conducted when infected snails were found, and study teams were hampered by the large number of water bodies and the difficulty reaching many of those identified as HWCS.

More than 4,000 intermediate host snails of *Bulinus truncatus* and *B. globosus* species were collected, and 60 of them were found to be shedding *Schistosoma* cercariae. The number of snails collected decreased over time. However, some snail population rebound occurred, noted mostly during the second visit after application of niclosamide. The species of schistosomes identified were *S. haematobium*, *Schistosoma bovis*, and *S. haematobium–S. bovis* hybrids. The significance of these hybrids for human health and future elimination efforts remains to be determined.^14^

#### Parasitologic results

**Figure 3.**
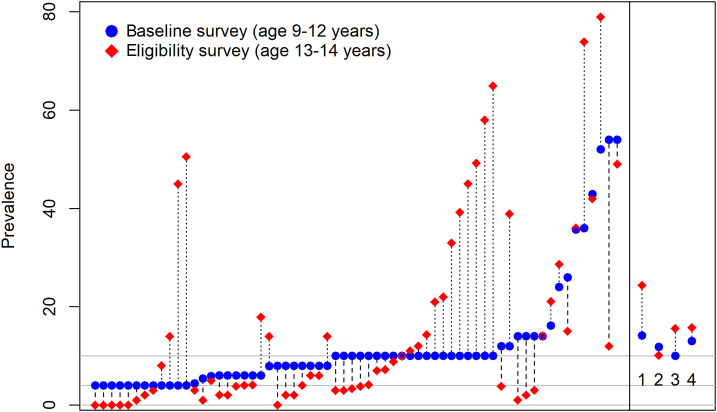
Comparison of prevalence in eligibility and baseline surveys, in 64 villages enrolled in the seasonal elimination study, Côte d’Ivoire. Each point on the *x* axis represents a single village. Arms 1–4 village mean for baseline and eligibility included on the right.

The baseline prevalence of *S. haematobium* in 9- to 12-year-old children was 24.8%, 10.1%, 13.9%, and 15.9%, in arms 1, 2, 3, and 4, respectively (Figure 3). These prevalences were higher than expected based on the eligibility survey results (Figure 3). As noted in Figure 3, there was a dramatic difference in the mean prevalence of arm 1 compared with the other three arms of the study. Reasons for this within-arm differences between eligibility and baseline testing are not clear, but such differences have been observed in other SCORE studies.^28^ They may relate to the children tested during the eligibility surveys being older or differences in the seasons during which testing was conducted.

The prevalence of schistosomiasis in 9- to 12-year-old children demonstrated statistically significant decreases from the baseline to the end of the study. Final prevalences were 7.5%, 3.5%, 0.6%, and 3.4% in arms 1, 2, 3, and 4, respectively. We also found a strong and important decline in the prevalence of infection in first-grade children and adults. Data are continuing to be analyzed, and more detailed results will be published in the near future.

### Test–treat–track–test–treat strategy.

#### Background.

Because MDA in places with very low prevalence means that most people who are treated are not infected, alternative, more targeted approaches have been suggested and attempted for other diseases such as malaria.^29,30^ For schistosomiasis, a T5 strategy could include ensuring that every suspected schistosomiasis case be tested, every confirmed case be treated with praziquantel, and the disease tracked through active surveillance that includes identifying and testing individuals who may share water exposure locations with index cases. The T5 strategy we are proposing for schistosomiasis involves testing classrooms of children, treating those who are positive, and interviewing and tracking them to identify others who are at risk because they are family or community members who share HWCS and are likely at risk. They would then be offered testing and treatment if needed.

Feasibility assessments of the T5 strategy were implemented in 2019 by SCORE investigators in Egypt, Kenya, and Tanzania. Schoolchildren who test positive by POC-CCA are treated, and a “second generation” of potentially exposed individuals—people living with a child testing positive and those frequenting the same HWCS as the index case—are identified and treated. Egyptian investigators have also reviewed and identified people testing positive in rural health facility records and tracking them to identify and offer testing to detect potential secondary cases. Because the T5 approach should be simple, easily implemented, acceptable, and of reasonable cost for it to be widely used, these evaluations include assessments of the costs, barriers, and difficulty of implementing the various steps. These data will be published in the near future.

### Modeling using SCORE data.

SCORE data have been used to model the potential impact of various interventions on *Schistosoma* infection prevalence and intensity and the likelihood of achieving elimination over a given period. For example, two groups, one based at Case Western Reserve University and the second based at Imperial College London, focused on schistosomiasis modeling in the NTD Modelling Consortium (NTDMC), funded by the Children’s Investment Fund and the BMGF.^31^

Data from SCORE studies and other study inputs were used by the two groups to develop and refine mathematical models for practical use in predicting long-term responses to various control strategies, and SCORE data from the gaining control study in Kenya^32,33^ have been used to calibrate models of persistent hot spot communities. As a first priority, the NTDMC examined whether implementation of MDA under the current WHO guidelines will achieve the 2020 goals from the WHO NTD road map, which are initial morbidity control (< 5% prevalence of heavy infections across sentinel sites) by 2020 and elimination as a public health problem (< 1% prevalence of heavy infections in sentinel sites) by 2025.

Both modeling groups found that the WHO goals could probably be consistently achieved in 6 years in low-prevalence settings (< 10% prevalence in school-aged children) but not in moderate- or high-prevalence settings.^34^ Potentially effective additional strategies include more frequent MDA, increasing coverage above 85% among children,^35^ the use of community wide treatment and other efforts to reach individuals who are persistently not treated, and addition of snail control.^36^ In the latter models, addition of snail control led to achievement of morbidity control in all settings. However, because of systematic noncompliance (i.e., people who repeatedly fail to receive treatment during MDAs), MDA without snail control will likely fail to achieve elimination as a public health problem in many villages. This is true even when MDA is increased to biannual in places that fail to reduce prevalence or intensity as expected (persistent hot spots, as determined after 3 years of school-based MDA).^37^

## SUMMARY

In most of sub-Saharan Africa, achieving the goal of interrupting transmission of schistosomiasis is likely to be several years away.^13^ Research and program evaluations, such as those conducted by SCORE, are critical in defining how best to move toward elimination in places that have achieved very low levels of prevalence and intensity.

In both SCORE elimination studies, interventions resulted in decreased prevalence and intensity of schistosomiasis among schoolchildren. Nevertheless, elimination of transmission was not achieved in either study. In Zanzibar, we were unable to demonstrate benefits from adding behavior change interventions or snail control to biennial MDA.

As in other SCORE studies, there were issues related to intervention implementation that could have impacted these results. These included implementation of MDA, implementation of behavior change interventions in Zanzibar, and implementation of snail control in both Zanzibar and Côte d’Ivoire.^17,18^

Achieving regular, high-quality MDA coverage can be challenging, impacted not only by NTD programmatic issues but also by events external to NTD programs, such as school schedules and political events. Developing and implementing behavior change programs with a high degree of community acceptance in Zanzibar took a long time and required more resources than were available. Achieving high coverage with niclosamide can also be resource-intensive and technically difficult. Thus, the absence of evidence that addition of snail control or a behavioral change intervention to biannual MDA was helpful in achieving elimination is not sufficient to conclude these interventions had no effect or that these are not useful adjuncts to MDA in efforts to achieve elimination and should not be cited as such.^38,39^ Mass drug administration compliance in Zanzibar and Côte d’Ivoire remained high, especially among children.^16^ Nevertheless, as the prevalence of schistosomiasis decreases and countries move toward elimination, building and sustaining political will to invest in schistosomiasis control and elimination may become more difficult in some places. During our studies on elimination on Zanzibar and in Côte d’Ivoire, political commitment remained high. Nevertheless, MDA in places with very low prevalence means that perhaps 90–95% of those treated are not infected. Alternative and more targeted approaches, such as the T5 approach being evaluated by SCORE, should be assessed in other places.^15^

A major limitation for both of the large intervention studies is the lack of a highly sensitive and specific test for S. *haematobium* that can be used cost-effectively on large numbers of samples. On Zanzibar, almost a third of the infected individuals had extremely light infection intensities based on urine filtration, with fewer than five eggs per 10 mL of urine.^40^ When using UCP-LF CAA assay results as a gold standard, urine filtration on Pemba had a sensitivity of 67% in children and 58% in adults. In areas on Pemba with an apparent prevalence of < 10% by urine filtration, the prevalence determined with the UCP-LF CAA assay was almost three times higher.^3,41^ Other limitations of the Zanzibar elimination study are described in a recent article on the 5-year study results.^16^

The SCORE studies provide lessons learned for future research, such as the difficulty conducting randomized control trials (RCTs) to assess strategies to interrupt transmission of schistosomiasis.^42^ Because cost considerations are likely to be important as countries move toward elimination, we suggest future research studies collect data prospectively, using specially designed tools, and ensure that staff who are collecting the data have adequate training and oversight, as discussed in another article in this supplement.^20^

The countries that have eliminated schistosomiasis have generally done so as socioeconomic conditions have dramatically improved, in concert with decades of public health interventions.^43^ The recent expansion of programs in water, sanitation, and hygiene presents an opportunity for mutual program benefit. Nevertheless, our studies on Zanzibar and in Côte d’Ivoire, as well as in the SCORE sustaining control studies in low-prevalence areas and the results of modeling indicate that schistosomiasis can be reduced further even where prevalence is already low.^31,34,44^

Although there remain several barriers to interrupting transmission of schistosomiasis as demonstrated in these SCORE studies and elsewhere,^13^ we remain optimistic that interrupting transmission of schistosomiasis will become an increasingly common goal and a reality. However, this will require continued investments in operational research to develop the needed tools and implement the adaptive strategies needed to finish the job.

## Supplemental file

Supplemental materials
