# Supplementary material for: SCORE Operational Research on Moving toward Interruption of Schistosomiasis Transmission
Source: Am J Trop Med Hyg. 2020 May 12;103(1 Suppl):58–65. doi: 10.4269/ajtmh.19-0825 (PMC7351301; doi:10.4269/ajtmh.19-0825)

Study and Implementation of

Schistosomiasis Elimination in Zanzibar (Unguja and Pemba Islands) Using an Integrated Multidisciplinary Approach

Standard Analyses Plan Results

Stefanie Knopp
Swiss Tropical and Public Health Institute
Basel
Switzerland

Jan Hattendorf
Swiss Tropical and Public Health Institute
Basel
Switzerland

David Rollinson
Natural History Museum
London
United Kingdom

and
Muriel Rabone
Natural History Museum
London
United Kingdom

12/10/2017

Figure 1a: Map of study schools and shehias by intervention arm: Pemba

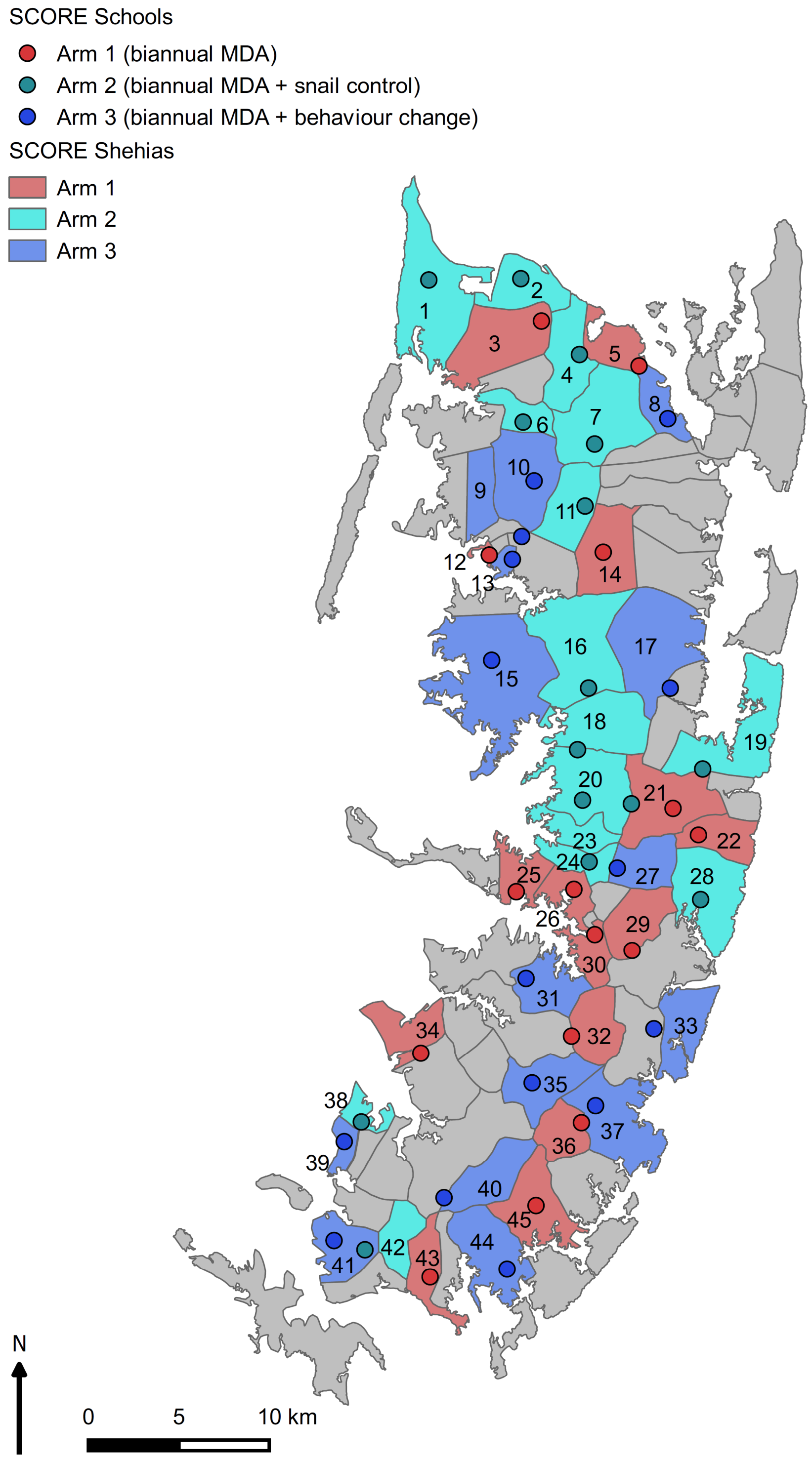

Shehia numbers:

1. Makangale; 2. Msuka; 3. Konde; 4. Kinowe; 5. Tumbe; 6. Mgogoni; 7. Shumba Viamboni; 8. Sizini; 9. Kizimbani; 10. Kinyasini; 11. Finya; 12. Selemu; 13. Jadida; 14. Pandani; 15. Mtambile Kusini; 16. Piki; 17. Mchangamdogo; 18. Kisiwani; 19. Kangagani; 20. Ziwani; 21. Ole; 22. Uwandani; 23. Mbuzini; 24. Kwale; 25. Wesha; 26. Tibrinzi; 27. Ng'ambwa; 28. Vitongoji; 29. Wawi; 30. Chanjaani; 31. Shungi; 32. Matale; 33. Pujini; 34. Wambaa; 35. Ngwachani; 36. Ukutini; 37. Chambani; 38. Makombeni; 39. Ng'ombeni; 40. Mtambile; 41. Michenzani; 42. Mkanyageni; 43. Kangani; 44. Kengeja; 45. Kiwani.

Figure 1a: Map of study schools and shehias by intervention arm: Unguja

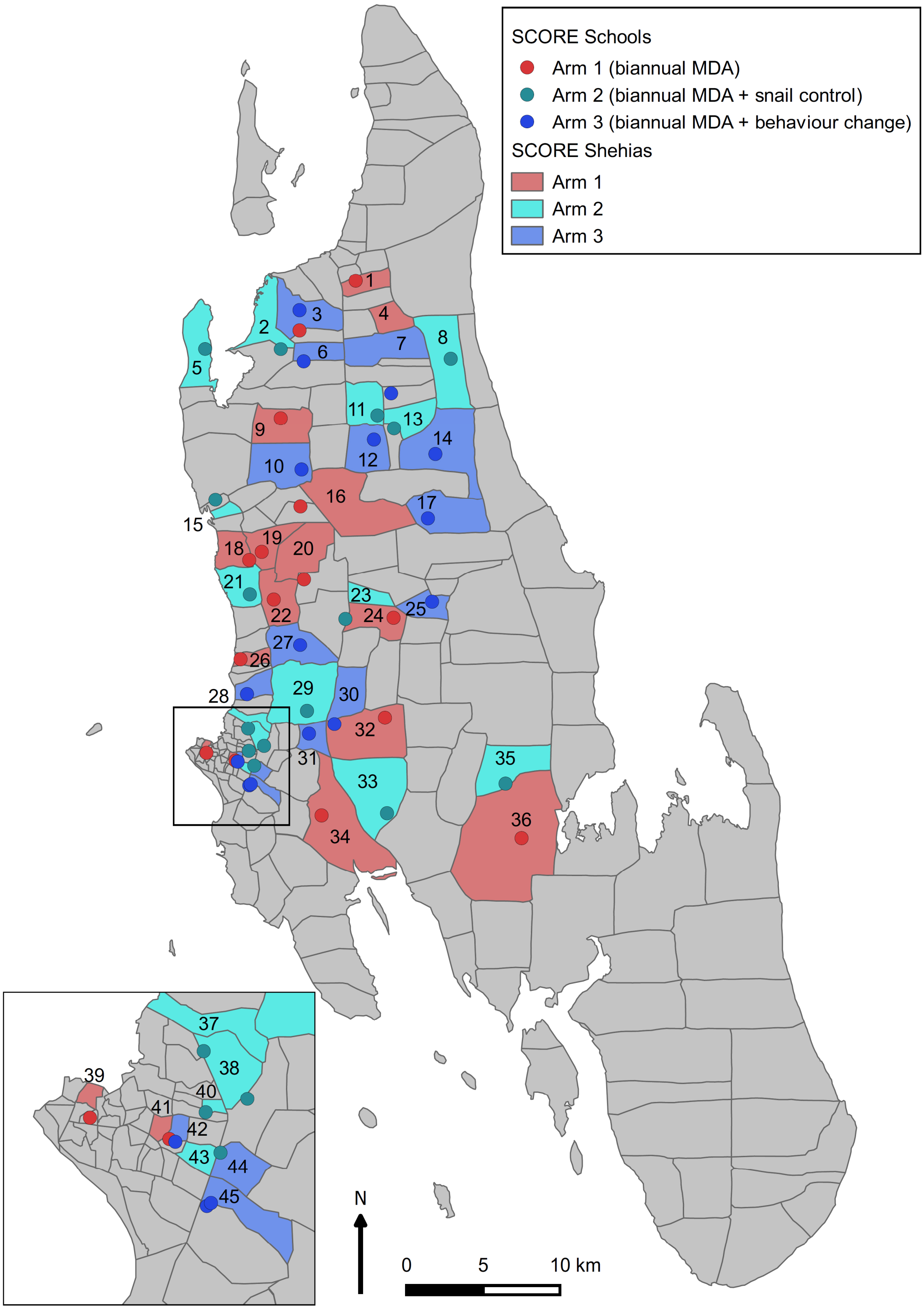

Shehia numbers:

1. Mkwajuni; 2. Donge Mchangani; 3. Pale; 4. Gamba; 5. Mafufuni (Kiongwe school); 6. Donge Mtambile; 7. Chaani Masingi; 8. Kandwi; 9 Donge Mnyimbi; 10. Mahonda ; 11. Bandamaji;12. Mgambo; 13. Kinyasini; 14. Upenja; 15. Fujoni; 16. Kitope;17. Kilombero; 18. Kama; 19. Mfenesini; 20. Mwakaje; 21. Chuini; 22. Mbuzini; 23. Miwani; 24. Kiboje Mkwajuni; 25. Uzini; 26. Mwanyanya (Bububu school); 27. Dole 28. Mtoni; 29. Kianga; 30. Koani; 31. Mwera(Regeza Mwendo school); 32. Ubago; 33. Jumbi; 34. Fuoni Kibondeni; 35. Jendele; 36. Cheju.

INDENT MAP: 37. Mtopepo; 38. Welezo; 39. Mchangani; 40. Kilimahewa Juu; 41. Muungano; 42. Sebleni; 43. Nyerere; 44. Mwanakwerekwe; 45. Melinne.

Figure 2: Flow diagram for progress of schools and individuals through ZEST cross-sectional studies (baseline in 2012 to Y6 in 2017)

Enrollment (2011)

Assessed for eligibility (n= 331 shehias)

Excluded (n= 241)

- Not meeting inclusion criteria (n=236)
- Randomized not to participate (n=5)

Randomized (n= 90 shehias); * please see protocol deviations

Randomization (2012)

Year	Arm	No. of schools (Baseline)	Number of participants per school	
			Mean	SD
1	**Zanzibar**			
	MDA	30	97.5	12.8
	Snail	29	97.2	17.3
	Behaviour	29	94.0	17.2
1	**Unguja**			
	MDA	15	98.8	16.8
	Snail	14*	102.5	12.4
	Behaviour	14**	97.9	18.7
1	**Pemba**			
	MDA	15	96.3	6.5
	Snail	15	91.2	19.8
	Behaviour	15	90.0	14.5

Follow-up (2012- 2016)

	Arm	No. of schools without data, by Year	No. of shehias that changed intervention arm or were moved, by Year, with reason
1	MDA	0	0
	Snail	1	0
	Behaviour	1	0
2	MDA	0	0
	Snail	0	0
	Behaviour	0	0
3	MDA	0	0
	Snail	0	0
	Behaviour	0	0
4	MDA	0	0
	Snail	0	0
	Behaviour	0	0
5	MDA	0	0
	Snail	0	0
	Behaviour	1	0
6	MDA	0	0
	Snail	0	0
	Behaviour	1	0

Y6 data (2017)

	Arm	No. of schools	Number of participants per school	
			Mean	SD
5	**Zanzibar**			
	MDA	30	108.3	11.7
	Snail	30	108.3	9.5
	Behaviour	29	108.5	11.5
5	**Unguja**			
	MDA	15	106.8	14.9
	Snail	15	107.7	7.8
	Behaviour	14***	107.0	15.3
5	Pemba			
	MDA	15	109.7	7.5
	Snail	15	108.8	11.0
	Behaviour	15	109.8	6.2

* Kiongwe school belongs to Mafufuni shehia, which was originally randomized, but the school was only surveyed from 2013 onwards. A wrong school, Makoba school in Makoba shehia, was surveyed instead in 2012. We keep Kiongwe school but not Makoba school in the primary analysis. Since no baseline data are available for Kiongwe, we only have 14 schools in the snail arm at baseline and 15 at follow-ups.

**Regeza Mwendo schools belongs to Mwera shehia, which was originally randomized, but the school was only surveyed from 2013 onwards. A wrong school, Machui school in Koani shehia, was surveyed instead in 2012. We keep Regeza Mwendo school but not Machui school in the primary analysis. Since no baseline data are available for Regeza Mwendo, we only have 14 schools in the snail arm at baseline and 15 at follow-ups.

***Mwanakerekwe B school in Mwanakerekwe shehia became a secondary school from 2016 onward; hence it was only surveyed from 2012-2015, but remains in primary analysis. Since no data are available for Regeza Mwendo in 2016 and 2017, we only have 14 schools in the behavior arm at the follow-ups in 2016 and 2017.

Note, in line with the email exchange with the SCORE secretariat on 06.10.2017:

Eligible to be included into the analyses were all children that met the following criteria:

```
consent==1 & standard!=1 & age>8 & age<13 & urine_comp==1 & school!="MAKOBA" & school!="KIBWENI" & school!="MACHUI"
```

urine_comp is defined as:

```
gen urine_comp=.
replace urine_comp=1 if haemastix_haem!=.
replace urine_comp=1 if uf_sh!=.
replace urine_comp=0 if haemastix_haem==. & uf_sh==.
```

A *S. haematobium*-positive result is defined as follows:

```
gen shpos=.
replace shpos=1 if uf_sh>0
replace shpos=1 if (uf_sh==. & haemastix_haem>0 & haemastix_haem!=.)
replace shpos=0 if (uf_sh== . & haemastix_haem == 0)
replace shpos=0 if uf_sh==0
replace shpos=. if  uf_sh==. & haemastix_haem==.
```

Hence, for all children that had no urine filtration result but a haemastix result, a microhaematuria result >0 was considered as *S. haematobium*-positive. The number of children with prevalence results is thus different from the number of children with intensity results.

Table 1a. Baseline characteristics of participants by study arm: <u>individual level</u> data summarized by study arm.

				9 ys		10 ys		11 ys		12 ys		*S. haematobium*							
Island	Study arm	Number of schools	Number of participants	F	M	F	M	F	M	F	M	Prevalence	Lower 95% CI	Upper 95% CI	Mean intensity per 10 ml	1st quartile	3rd quartile	Number of children with positive egg counts	Mean intensity of positive counts only
Zanzibar	MDA	30	2853	273	155	609	446	452	367	218	333	4.2	3.5	4.9	2.8	0	0	118	68.0
Zanzibar	Snail	29	2688	279	149	605	365	320	395	218	357	7.8	6.8	8.8	5.7	0	0	207	73.5
Zanzibar	Behaviour	29	2613	269	167	514	404	371	331	243	314	6.4	5.5	7.3	5.3	0	0	163	84.6
Pemba	MDA	15	1437	110	50	311	218	266	198	123	161	4.9	3.8	6.0	5.0	0	0	70	103.0
Pemba	Snail	15	1276	105	45	272	155	163	197	129	210	11.1	9.3	12.8	10.2	0	0	141	92.4
Pemba	Behaviour	15	1304	109	75	243	184	210	165	150	168	8.9	7.4	10.4	9.6	0	0	115	108.1
Unguja	MDA	15	1416	163	105	298	228	186	169	95	172	3.5	2.5	4.4	0.6	0	0	48	16.8
Unguja	Snail	14	1412	174	104	333	210	157	198	89	147	4.8	3.7	5.9	1.6	0	0	66	33.1
Unguja	Behaviour	14	1309	160	92	271	220	161	166	93	146	3.9	2.8	5.0	1.0	0	0	48	28.1

Table 1b. Baseline characteristics of participants by study arm: <u>village level</u> results summarized by study arm.

				Prevalence						Intensity (eggs per 10 ml)						Intensity for positive counts only (eggs per 10 ml)					
Island	Study arm	Number of schools	Number of participants	Min	1st Qu	Median	Mean	3rd Qu	Max	Min	1st Qu	Median	Mean	3rd Qu	Max	Min	1st Qu	Median	Mean	3rd Qu	Max
Zanzibar	MDA	30	2853	0.0	1.1	3.2	4.1	4.8	21.8	0.0	0.0	0.3	2.8	1.3	36.6	1.0	5.0	10.3	46.7	33.3	247.8
Zanzibar	Snail	29	2688	0.0	1.9	4.8	8.1	13.5	24.2	0.0	0.1	0.6	6.3	27.1	27.1	1.0	9.0	30.5	61.7	87.7	257.6
Zanzibar	Behaviour	29	1613	0.0	0.0	2.6	6.1	9.2	29.6	0.0	0.0	0.2	5.0	2.6	45.6	1.0	7.8	21.8	49.9	98.4	156.5
Pemba	MDA	15	1437	0.0	1.2	3.9	5.0	5.0	21.8	0.0	0.1	0.5	5.0	4.8	36.6	3.0	5.5	16.4	68.3	120.0	247.8
Pemba	Snail	15	1276	0.0	3.6	10.1	11.1	18.6	24.2	0.0	0.5	7.8	10.6	22.0	27.1	6.5	21.5	60.8	86.9	131.6	257.6
Pemba	Behaviour	15	1304	0.0	1.1	2.9	8.3	10.7	29.6	0.0	0.0	1.6	8.7	14.8	45.6	1.0	14.0	28.5	63.2	125.0	156.5
Unguja	MDA	15	1416	0.0	0.0	3.0	3.3	4.7	13.0	0.0	0.0	0.2	0.6	0.4	4.4	1.0	3.0	8.2	19.2	11.3	130.7
Unguja	Snail	14	1412	0.0	1.6	2.0	4.8	4.8	18.9	0.0	0.0	0.2	1.6	3.8	7.7	1.0	1.5	9.0	29.6	36.6	168.3
Unguja	Behaviour	14	1309	0.0	0.0	1.1	3.8	7.0	16.7	0.0	0.0	0.0	0.9	0.8	5.4	2.2	2.4	16.0	25.3	43.7	85.3

Kiongwe school belongs to Mafufuni shehia, which was originally randomized, but the school was only surveyed from 2013 onwards. A wrong school, Makoba school in Makoba shehia, was surveyed instead in 2012. We keep Kiongwe school but not Makoba school in the primary analysis. Since no baseline data are available for Kiongwe, we only have **14 schools** in the snail arm at baseline and 15 at follow-ups.

Regeza Mwendo schools belongs to Mwera shehia, which was originally randomized, but the school was only surveyed from 2013 onwards. A wrong school, Machui school in Koani shehia, was surveyed instead in 2012. We keep Regeza Mwendo school but not Machui school in the primary analysis. Since no baseline data are available for Regeza Mwendo, we only have **14 schools** in the snail arm at baseline and 15 at follow-ups.

Table 2a. Community wide and school based treatment coverage: Zanzibar (both islands)

Year	Study arm	MDA Round	Number of shehias with schools (MoH data available)	Number of school children registered in school (MoH)	Number of school children treated (MoH)	% school children treated (MoH)	Number of school children surveyed (SCORE post-MDA survey)	Number of school children treated (SCORE post-MDA survey)	% school children treated (SCORE post-MDA survey)	Total population (national census)	Number of shehias (MoH data available)	Total population (MoH)	Total population treated (MoH)	Total population eligible for treatment (MoH)	% total population treated (MoH)	Number of adults surveyed (SCORE post-MDA survey)	Adults received treatment (SCORE post-MDA survey)	Adults complied with treatment (swallowed all tablets together) (SCORE post-MDA survey)	% Adults complying (SCORE post-MDA survey)
2012		1																	
	MDA									137379	31	116746	99187		85.0				
	Snail									137841	31	118596	97744		82.4				
	Behaviour									152548	30	137953	105450		76.4				
		2																	
	MDA										31	147511	122100		82.8				
	Snail										31	127429	104634		82.1				
	Behaviour										30	136698	118409		86.6				
2013		3																	
	MDA									141225.61	30	121089	88261	99511	72.9				
	Snail									141700.55	31	119681	91732	104918	76.6				
	Behaviour									156819.35	30	118809	87221	101752	73.4				
		4																	
	MDA		25	18022	13011	72.2	3221	2368	73.5		31	151775	79993	95440	52.7	1631	996	812	49.8
	Snail		23	13007	9748	74.9	3262	2276	69.8		31	128311	88621	112615	69.1	1489	874	719	48.3
	Behaviour		24	25289	19220	76.0	3164	2535	80.1		30	127390	88512	108168	69.5	1434	851	712	49.7
2014		5																	
	MDA									145179.93	31	128381	84837	100029	66.1				
	Snail									145668.16	31	135072	96332	115553	71.3				
	Behaviour									161210.28	30	139888	91931	116218	65.7				
		6																	
	MDA		15	13023	11155	85.7	3276	2661	81.2		15	61932	40419	47644	65.3	1688	1186	916	54.3
	Snail		14	9775	7457	76.3	3365	2702	80.3		15	103447	65950	80318	63.8	1490	1050	783	52.6
	Behaviour		15	17433	14058	80.6	3230	2709	83.9		15	66864	45481	56290	68.0	1438	1023	712	49.5
2015		7																	
	MDA		31	37374	31454	84.2				149244.97	31	123317	68236	88501	55.3				
	Snail		31	40969	30999	75.7				149746.87	31	138000	83869	109957	60.8				
	Behaviour		29	40653	32045	78.8				165724.18	30	129842	75649	98539	58.3				
		8																	
	MDA		31	49360	36810	74.6	3298	3052	92.5		31	106996	73832	93540	69.0	1691	1019	740	43.8
	Snail		31	43405	32948	75.9	3398	3172	93.3		31	126034	89604	114197	71.1	1493	914	682	45.7
	Behaviour		30	47702	35818	75.1	3114	2872	92.2		30	114038	79227	101873	69.5	1438	895	611	42.5
2016		9																	
	MDA		31	40233	35033	87.1				153423.83	31	120178	48779	84304	40.6				
	Snail		31	42653	35995	84.4				153939.78	31	128345	45045	90304	35.1				
	Behaviour		30	50930	42862	84.2				170364.45	30	115166	49222	83809	42.7				
		10																	
	MDA		30	42545	38430	90.3	3192	3126	97.9		31	121190	74976	90234	61.9				
	Snail		30	45660	39098	85.6	3236	3158	97.6		30	138817	89277	107045	64.3				
	Behaviour		30	51386	46469	90.4	3093	3034	98.1		30	134410	79978	95689	59.5				

Table 2b. Community wide and school based treatment coverage: Pemba

Year	Study arm	MDA Round	Number of shehias with schools (MoH data available)	Number of school children registered in school (MoH)	Number of school children treated (MoH)	% school children treated (MoH)	Number of school children surveyed (SCORE post-MDA survey)	Number of school children treated (SCORE post-MDA survey)	% school children treated (SCORE post-MDA survey)	Total population (national census)	Number of shehias (MoH data available)	Total population (MoH)	Total population treated (MoH)	Total population eligible for treatment (MoH)	% total population treated (MoH)	Number of adults surveyed (SCORE post-MDA survey)	Adults received treatment (SCORE post-MDA survey)	Adults complied with treatment (swallowed all tablets together) (SCORE post-MDA survey)	% Adults complying (SCORE post-MDA survey)
2012		1																	
	MDA									69439	16	67562	56076		83.0				
	Snail									53127	16	52439	43946		83.8				
	Behaviour									67452	15	67062	55241		82.4				
		2																	
	MDA										16	79358	68059		85.8				
	Snail										16	50681	42331		83.5				
	Behaviour										15	67011	55690		83.1				
2013		3																	
	MDA									71383	16	67134	50150	55120	74.7				
	Snail									54615	16	48984	38079	42533	77.7				
	Behaviour									69341	15	64209	50558	56344	78.7				
		4																	
	MDA		15	10821	9472	87.5	1755	1538	87.6		16	102183	46890	56452	45.9	740	405	323	43.6
	Snail		15	7299	6129	84.0	1712	1432	83.6		16	54839	37912	47355	69.1	746	403	328	44.0
	Behaviour		13	10115	8206	81.1	1643	1400	85.2		15	67496	47673	57790	70.6	745	392	328	44.0
2014		5																	
	MDA									73382	16	68514	46328	55378	67.6				
	Snail									56144	16	56145	37432	46438	66.7				
	Behaviour									71282	15	73669	49507	61467	67.2				
		6																	
	MDA		15	13023	11155	85.7	1725	1625	94.2		16	Health post	17443	NA	NA	744	508	367	49.3
	Snail		14	9775	7457	76.3	1695	1595	94.1		16	Health post	13145	NA	NA	741	500	350	47.2
	Behaviour		15	17433	14058	80.6	1715	1600	93.3		15	Health post	22955	NA	NA	742	496	312	42.0
2015		7																	
	MDA		16	21400	17038	79.6				75437	16	78810	36543	50154	46.4				
	Snail		16	20743	15033	72.5				57716	16	58918	28314	40724	48.1				
	Behaviour		15	21811	15720	72.1				73278	15	65030	33786	46004	52.0				
		8																	
	MDA		16	31072	21659	69.7	1649	1526	92.5		16	52314	37738	45612	72.1	745	405	242	32.5
	Snail		16	18966	14473	76.3	1699	1586	93.3		16	43534	32005	42808	73.5	745	404	257	34.5
	Behaviour		15	30199	22083	73.1	1557	1436	92.2		15	48057	34289	39446	71.4	742	428	228	30.7
2016		9																	
	MDA		16	22215	19482	87.7				77549	16	68665	29050	37227	42.3				
	Snail		16	21430	17392	81.2				59332	16	43662	21887	28629	50.1				
	Behaviour		15	26445	22681	85.8				75330	15	58764	27540	34014	46.9				
		10																	
	MDA		16	25745	23391	90.9	1644	1614	98.2		16	66143	33404	40024	50.5				
	Snail		16	24992	21748	87.0	1627	1596	98.1		16	54863	29999	34859	54.7				
	Behaviour		15	31219	29217	93.6	1654	1634	98.8		15	71686	36021	40903	50.2				

Issues and concerns related to MDA coverage: Pemba

- SBT was only introduced in 2013, in round 4. It was not conducted in 2014, round 5, due to a lack of funds. Subsequently, SBT was added to CWT in round 6, 7, 8, 9, and 10.
- In 2013, SBT round 4, behavior arm: no MoH SBT data were available from Shungi and Mchangamdodo, hence we show data from 13shehias only.
- In 2014, SBT round 6, snail arm: no MoH SBT data were available from Makanagale, hence we show data from 14 shehias only.
- In 2015-2016, SBT rounds 7-10, snail arm: Tumbe shehia was split in Tumbe East and Tumbe West. Hence we show SBT data from 16 shehias.
- In 2015-2016, SBT rounds 7-10, behaviour arm: Msuka shehia was split in Msuka East and Msuka West. Hence we show SBT data from 16 shehias.
- In 2012, a national census was conducted. The annual growth factor is indicated at 2.8. Hence, we are able to calculate population numbers for the subsequent years.
- In 2012, in CWT round 1, it is not clear whether the population recorded by the CDDs is the total or eligible population. It is not differentiated by sex, adult or school-aged population.
- In 2012, in CWT round 2, it is mentioned that the total population is the total population in the households. It is not differentiated by sex, adult or school-aged population.
- In 2014, in CWT round 6, health posts instead of CDDs were implemented. No coverage was assessed.
- In all CWT rounds implemented from 2012-2017:
    - Tumbe shehia was split in Tumbe East and Tumbe West; MoH coverage data include both (➔ 16 shehias)
    - Msuka shehia was split in Msuka East and Msuka West; MoH coverage data include both (➔ 16 shehias)
- General: Pujini shehia was split in Dodo and Kumvini; MoH coverage data were combined as Pujini.
- Post-MDA surveys were conducted within the SCORE parasitology surveys in 2014, 2015 and 2016, hence for round 4, 6 and 8.
- In 2014, in SBT round 4, schools in Chanjaani shehia have more children treated than children registered, hence the coverage is >100% (MoH data).
- In 2015, in SBT round 6, schools in Shungi and Kiwanie shehias have more children treated than children registered, hence the coverage is >100% (MoH data).
- In 2016, in CWT round 10, Kisiwani shehia has more people treated than total population, hence the coverage is >100% (MoH data).
- In 2016, SBT round 10, schools in Konde, Chambani, Kangani, Kiwani, Mtambile, Ngombeni, Ukutini shehias have more children treated than children registered (MoH data).

Table 2c. Community wide and school based treatment coverage: Unguja

Year	Study arm	MDA Round	Number of shehias with schools (MoH data available)	Number of school children registered in school (MoH)	Number of school children treated (MoH)	% school children treated (MoH)	Number of school children surveyed (SCORE post-MDA survey)	Number of school children treated (SCORE post-MDA survey)	% school children treated (SCORE post-MDA survey)	Total population (national census)	Number of shehias (MoH data available)	Total population (MoH)	Total population treated (MoH)	Total population eligible for treatment (MoH)	% total population treated (MoH)	Number of adults surveyed (SCORE post-MDA survey)	Adults received treatment (SCORE post-MDA survey)	Adults complied with treatment (swallowed all tablets together) (SCORE post-MDA survey)	% Adults complying (SCORE post-MDA survey)
2012		1																	
	MDA									67940	15	49184	43111		87.7				
	Snail									84714	15	66157	53798		81.3				
	Behaviour									85096	15	70891	50209		70.8				
		2																	
	MDA										15	68153	54041		79.3				
	Snail										15	76748	62303		81.2				
	Behaviour										15	69687	62719		90.0				
2013		3																	
	MDA									69842	14	53955	38111	44391	70.6				
	Snail									87086	15	70697	53653	62385	75.9				
	Behaviour									87479	15	54600	36663	45408	67.1				
		4																	
	MDA		10	7201	3539	49.1	1466	830	56.6		15	49592	33103	38988	66.8	891	591	489	54.9
	Snail		8	5708	3619	63.4	1550	844	54.5		15	73472	50709	65260	69.0	743	471	391	52.6
	Behaviour		11	15174	11014	72.6	1521	1135	74.6		15	59894	40839	50378	68.2	689	459	384	55.7
2014		5																	
	MDA									71798	15	59867	38509	44651	64.3				
	Snail									89524	15	78927	58900	69115	74.6				
	Behaviour									89928	15	66219	42424	54751	64.1				
		6																	
	MDA						1551	1036	66.8		15	61932	40419	47644	65.3	944	678	549	58.2
	Snail						1670	1107	66.3		15	103447	65950	80318	63.8	749	550	433	57.8
	Behaviour						1515	1109	73.2		15	66864	45481	56290	68.0	696	527	400	57.5
2015		7																	
	MDA		15	15974	14416	90.2				73808	15	44507	31693	38347	71.2				
	Snail		15	20226	15966	78.9				92031	15	79082	55555	69233	70.2				
	Behaviour		14	18842	16325	86.6				92446	15	64812	41863	52535	64.6				
		8																	
	MDA		15	18288	15151	82.8	1649	1526	92.5		15	54682	36094	47928	66.0	946	614	498	52.6
	Snail		15	24439	18475	75.6	1699	1586	93.3		15	82500	57599	71389	69.8	748	510	425	56.8
	Behaviour		15	17503	13735	78.5	1557	1436	92.2		15	65981	44938	62427	68.1	696	467	383	55.0
2016		9																	
	MDA		15	18018	15551	86.3				75875	15	51513	19729	47077	38.3				
	Snail		15	21223	18603	87.7				94608	15	84683	23158	61675	27.3				
	Behaviour		15	24485	20181	82.4				95035	15	56402	21682	49795	38.4				
		10																	
	MDA		14	16800	15039	89.5	1548	1512	97.7		15	55047	41572	50210	75.5				
	Snail		14	20668	17350	83.9	1609	1562	97.1		14	83954	59278	72186	70.6				
	Behaviour		15	20167	17252	85.5	1439	1400	97.3		15	62724	43957	54786	70.1				

Issues and concerns related to MDA coverage: Unguja

- MoH data for 2016 had not yet been submitted by the end of Feb, 2017. Hence, no data are yet shown for rounds 9 and 10.
- SBT was only introduced in 2013, in round 4. It was not conducted in 2014, rounds 5 and 6, due to a lack of funds. Subsequently, SBT was added to CWT in rounds 7, 8, 9, and 10.
- In 2013, in SBT round 4, no data are recorded for the schools in the following shehias (MoH data):
    - MDA arm: Kiboje, Mkwajuni, Muungano, Mwakaje, Ubago. Hence, we show results for schools from 10 shehias.
    - Snail arm: Chuini, Donge Mchangani, Jendele, Jumbi, Kilimahew Juu, Mtopepo, Nyerere. Hence, we show results for schools from 8 shehias.
    - Behaviour arm: Dole, Melinne, Pale, Sebleni. Hence, we show results for schools from 11 shehias.
- The SCORE coverage survey conducted in 2014 for SBT round 4 revealed that children visiting Std 3 and Std 4 in the following schools did not receive treatment
    - MDA arm: Kiboje, Mkwajuni, Muungano, Ubago
    - Snail arm: Jendele, Jumbi, Kilimahewa B, Nyerere
    - Behaviour arm: Mwanakerekwe H/Melinne, Pale, Sebleni
- In 2015, in SBT round 7, different data were recorded for Upenja school. Hence, data for Upenja were excluded (MoH data). Hence, we show results for schools from 14 shehias.
- In 2012, in CWT round 1 and CWT round 2, it is not clear whether the population recorded by the CDDs is the total or eligible population. It is not differentiated by sex, adult or school-aged population.
- In 2013, in CWT round 4, no data are recorded for Gamba shehia (MoH data).
- In 2016, in CWT round 10, no data are recorded for Miwani shehia (MoH data).
- In 2014, CWT round 4, Miwani shehia has more people treated than total population, hence the coverage is >100% (MoH data).
- In 2015, in CWT round 7, Fujoni shehia has more people treated than total population, hence the coverage is >100% (MoH data).
- In 2016, in CWT round 10, Kama and Mwakaje shehias hav more people treated than total population.

Details on method of collecting demographic data, both islands

The Ministry of Health (MoH) data were collected in the frame of community wide treatment (CWT) by community drug distributors (CDDs). CDDs followed a door to door approach registering the people living in the households of their responsibility and recording the number of people they provided drugs with. The approach was not directly observed treatment (DOT). Summary data for each shehia were provided to the MoH.

The MoH data were collected in the frame of school based treatment (SBT) by teachers, who recorded the number and sex of children registered in school and the number and sex of treated children. Summary data for each school were provided to the MoH.

In 2012, a national census was conducted. The annual growth factor is indicated at 2.8. Hence, we are able to calculate population numbers for the subsequent years.

The SCORE post-MDA surveys with questionnaires on drug compliance were conducted within the frame of the parasitology surveys, as described in the study protocol.

Listing of serious adverse events or harms, both islands:

- 2012, round 1: No single case of severe reaction, which needed hospitalization, was reported.

- 2012, round 2: No case of severe reaction, which needed hospitalization, was reported. Cases of nausea and vomiting were observed in some areas.
- 2013, round 3: No major adverse reactions were reported in any area. All reported cases were simple and most of them were due to nausea and vomiting and some few cases experienced body weakness.
- 2013, round 4: Two cases of severe reactions, which needed hospitalisation, were reported: one from each island. In Unguja, a girl of 13 years old developed body weakness and was hospitalised at Kivunge Hospital for six hours for IV fluid, then discharged, but remained weak and unable to walk without assistance for two weeks, then she recovered fully. Also a girl of 16 years from Pemba was hospitalised at Chake Chake Hospital and then referred to Mnazi Mmoja Hospital following severe swelling of both eye lids. The diagnosis at Mnazi Mmoja was Cavernous Thrombosis. The patient was referred to Muhimbili Hospital in Dar es Salaam, where the final diagnosis was a tumour.
- 2014, round 5: No major adverse reactions were reported in any area. All reported cases were simple and most of them were due to nausea and vomiting and some few cases experienced body weakness.
- 2014, round 6: No major adverse reactions were reported in any area. All reported cases were simple and most of them were due to nausea and vomiting and some few cases experienced body weakness.
- 2015, round 7: No case of severe reaction, which needed hospitalization, was reported. Cases of nausea and vomiting were observed in some areas.
- 2015, round 8: No adverse events mentioned in report.
- 2016, round 9: No major adverse reactions needing hospitalization were reported in any area. About 33 cases were reported, 13 in Pemba and 20 in Unguja, all with abdominal symptoms such as nausea, vomiting and some few cases experiencing body weakness.
- 2016, round 10: no case of severe reaction which needed hospitalization, was reported. However, cases of nausea and vomiting were common in most areas, especially among school children. The most fear observed reaction, which is a challenge to the programme and general population is the development of body weakness plus minus loss of consciousness. Three cases have been observed in Unguja and two in Pemba during this round.

Table 3. Descriptive results for baseline (2012) and year 6 (2017).

Overall

Zanzibar	Arm 1: MDA	Arm 2: Snail	Arm 3: Behaviour
Number tested at baseline	2853	2688	2613
Number infected at baseline	120	209	167
Prevalence at baseline	4.2	7.8	6.4
Number tested in Year 6	3184	3217	3080
Number infected in Year 6	46	56	58
Prevalence in Year 6	1.4	1.7	1.9
Absolute difference between prevalence at Year 6 and baseline	-2.8	-6.0	-4.5
Relative difference between prevalence in Year 6 and baseline (% change)	-65.7	-77.6	-70.5
Village level arithmetic mean infection intensity at baseline (including zeros)	2.8	6.3	5.0
Village level arithmetic mean infection intensity at Year 6 (including zeros)	1.0	1.0	1.5
Egg reduction rate (1- Year 6 intensity/baseline)	0.6	0.8	0.7
Individual-level aritmetic mean infection intensity at baseline (without zeros)	68.0	73.5	84.6
Individual-level aritmetic mean infection intensity at Year 6 (without zeros)	75.4	58.5	78.4
**Pemba**	**Arm 1: MDA**	**Arm 2: Snail**	**Arm 3: Behaviour**
Number tested at baseline	1437	1276	1304
Number infected at baseline	71	141	116
Prevalence at baseline	4.9	11.1	8.9
Number tested in Year 6	1637	1610	1641
Number infected in Year 6	25	23	34
Prevalence in Year 6	1.5	1.4	2.1
Absolute difference between prevalence at Year 6 and baseline	-3.4	-9.6	-6.8
Relative difference between prevalence in Year 6 and baseline (% change)	-69.1	-87.1	-76.7
Village level arithmetic mean infection intensity at baseline (including zeros)	5.0	10.6	8.7
Village level arithmetic mean infection intensity at Year 6 (including zeros)	1.6	1.1	2.1
Egg reduction rate (1- Year 6 intensity/baseline)	0.7	0.9	0.8
Individual-level aritmetic mean infection intensity at baseline (without zeros)	103.0	92.4	108.1
Individual-level aritmetic mean infection intensity at Year 6 (without zeros)	108.1	79.9	107.2
**Unguja**	**Arm 1: MDA**	**Arm 2: Snail**	**Arm 3: Behaviour**
Number tested at baseline	1367	1344	1258
Number infected at baseline	49	68	51
Prevalence at baseline	3.6	5.1	4.1
Number tested in Year 6	1547	1607	1439
Number infected in Year 6	21	33	24
Prevalence in Year 6	1.4	2.1	1.7
Absolute difference between prevalence at Year 6 and baseline	-2.2	-3.0	-2.4
Relative difference between prevalence in Year 6 and baseline (% change)	-62.1	-59.4	-58.9
Village level arithmetic mean infection intensity at baseline (including zeros)	0.6	1.6	0.9
Village level arithmetic mean infection intensity at Year 6 (including zeros)	0.5	0.8	0.8
Egg reduction rate (1- Year 6 intensity/baseline)	0.2	0.5	0.1
Individual-level aritmetic mean infection intensity at baseline (without zeros)	16.8	33.1	28.1
Individual-level aritmetic mean infection intensity at Year 6 (without zeros)	36.6	43.6	37.6

Girls

Zanzibar	Arm 1: MDA	Arm 2: Snail	Arm 3: Behaviour
Number tested at baseline	1552	1422	1397
Number infected at baseline	41	80	75
Prevalence at baseline	2.6	5.6	5.4
Number tested in Year 6	1603	1696	1580
Number infected in Year 6	20	18	15
Prevalence in Year 6	1.2	1.1	0.9
Absolute difference between prevalence at Year 6 and baseline	-1.4	-4.6	-4.4
Relative difference between prevalence in Year 6 and baseline (% change)	-52.8	-81.1	-82.3
Village level arithmetic mean infection intensity at baseline (including zeros)	0.5	3.1	1.9
Village level arithmetic mean infection intensity at Year 6 (including zeros)	0.5	0.9	1.4
Egg reduction rate (1- Year 6 intensity/baseline)	-0.1	0.7	0.3
Individual-level aritmetic mean infection intensity at baseline (without zeros)	18.8	58.1	41.0
Individual-level aritmetic mean infection intensity at Year 6 (without zeros)	43.6	93.9	128.1
**Pemba**	**Arm 1: MDA**	**Arm 2: Snail**	**Arm 3: Behaviour**
Number tested at baseline	810	669	712
Number infected at baseline	20	51	55
Prevalence at baseline	2.5	7.6	7.7
Number tested in Year 6	835	862	850
Number infected in Year 6	10	8	11
Prevalence in Year 6	1.2	0.9	1.3
Absolute difference between prevalence at Year 6 and baseline	-1.3	-6.7	-6.4
Relative difference between prevalence in Year 6 and baseline (% change)	-51.5	-87.8	-83.2
Village level arithmetic mean infection intensity at baseline (including zeros)	0.8	5.2	3.3
Village level arithmetic mean infection intensity at Year 6 (including zeros)	0.3	0.6	1.8
Egg reduction rate (1- Year 6 intensity/baseline)	0.7	0.9	0.5
Individual-level aritmetic mean infection intensity at baseline (without zeros)	29.9	78.5	49.4
Individual-level aritmetic mean infection intensity at Year 6 (without zeros)	21.8	76.1	144.4
**Unguja**	**Arm 1: MDA**	**Arm 2: Snail**	**Arm 3: Behaviour**
Number tested at baseline	742	753	685
Number infected at baseline	21	29	20
Prevalence at baseline	2.8	3.9	2.9
Number tested in Year 6	768	834	730
Number infected in Year 6	10	10	4
Prevalence in Year 6	1.3	1.2	0.5
Absolute difference between prevalence at Year 6 and baseline	-1.5	-2.7	-2.4
Relative difference between prevalence in Year 6 and baseline (% change)	-54.0	-68.9	-81.2
Village level arithmetic mean infection intensity at baseline (including zeros)	0.2	0.9	0.4
Village level arithmetic mean infection intensity at Year 6 (including zeros)	0.8	1.2	0.9
Egg reduction rate (1- Year 6 intensity/baseline)	-2.6	-0.4	-1.4
Individual-level aritmetic mean infection intensity at baseline (without zeros)	8.2	22.2	15.3
Individual-level aritmetic mean infection intensity at Year 6 (without zeros)	65.4	108.1	83.5

Boys

Zanzibar	Arm 1: MDA	Arm 2: Snail	Arm 3: Behaviour
Number tested at baseline	1301	1266	1216
Number infected at baseline	79	129	92
Prevalence at baseline	6.1	10.2	7.6
Number tested in Year 6	1581	1521	1500
Number infected in Year 6	26	38	43
Prevalence in Year 6	1.6	2.5	2.9
Absolute difference between prevalence at Year 6 and baseline	-4.4	-7.7	-4.7
Relative difference between prevalence in Year 6 and baseline (% change)	-72.9	-75.5	-62.1
Village level arithmetic mean infection intensity at baseline (including zeros)	5.4	10.5	9.0
Village level arithmetic mean infection intensity at Year 6 (including zeros)	1.6	1.0	1.6
Egg reduction rate (1- Year 6 intensity/baseline)	0.7	0.9	0.8
Individual-level aritmetic mean infection intensity at baseline (without zeros)	94.1	83.2	119.9
Individual-level aritmetic mean infection intensity at Year 6 (without zeros)	99.9	41.7	61.0
**Pemba**	**Arm 1: MDA**	**Arm 2: Snail**	**Arm 3: Behaviour**
Number tested at baseline	627	607	592
Number infected at baseline	51	90	61
Prevalence at baseline	8.1	14.8	10.3
Number tested in Year 6	802	748	791
Number infected in Year 6	15	15	23
Prevalence in Year 6	1.9	2.0	2.9
Absolute difference between prevalence at Year 6 and baseline	-6.3	-12.8	-7.4
Relative difference between prevalence in Year 6 and baseline (% change)	-77.0	-86.5	-71.8
Village level arithmetic mean infection intensity at baseline (including zeros)	9.8	17.9	16.1
Village level arithmetic mean infection intensity at Year 6 (including zeros)	3.0	1.5	2.5
Egg reduction rate (1- Year 6 intensity/baseline)	0.7	0.9	0.8
Individual-level aritmetic mean infection intensity at baseline (without zeros)	132.3	100.3	162.0
Individual-level aritmetic mean infection intensity at Year 6 (without zeros)	165.6	81.9	89.4
**Unguja**	**Arm 1: MDA**	**Arm 2: Snail**	**Arm 3: Behaviour**
Number tested at baseline	674	659	624
Number infected at baseline	28	39	31
Prevalence at baseline	4.2	5.9	5.0
Number tested in Year 6	779	773	709
Number infected in Year 6	11	23	20
Prevalence in Year 6	1.4	3.0	2.8
Absolute difference between prevalence at Year 6 and baseline	-2.7	-2.9	-2.1
Relative difference between prevalence in Year 6 and baseline (% change)	-66.0	-49.7	-43.2
Village level arithmetic mean infection intensity at baseline (including zeros)	1.0	2.6	1.5
Village level arithmetic mean infection intensity at Year 6 (including zeros)	0.1	0.4	0.7
Egg reduction rate (1- Year 6 intensity/baseline)	0.9	0.8	0.5
Individual-level aritmetic mean infection intensity at baseline (without zeros)	23.6	41.7	35.8
Individual-level aritmetic mean infection intensity at Year 6 (without zeros)	10.4	15.5	28.5

Figure 3a. Intensity categories by arm by year: Zanzibar (1=MDA Arm, 2=Snail Arm, 3=Behaviour Arm)

Overall

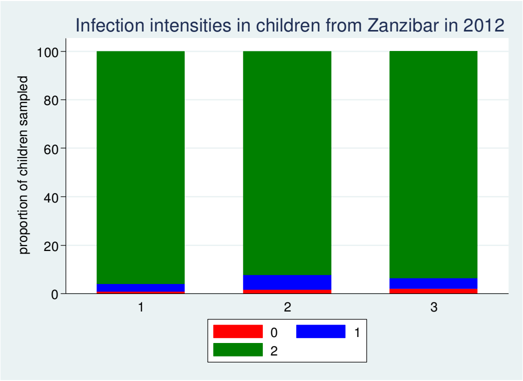

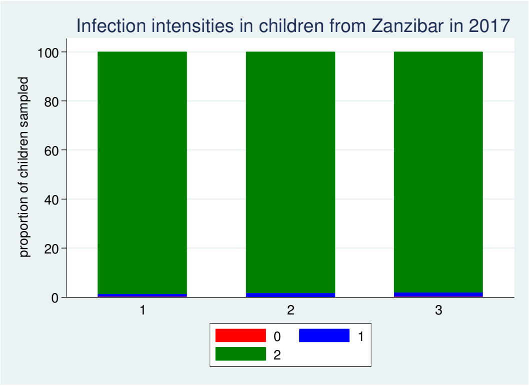

Girls

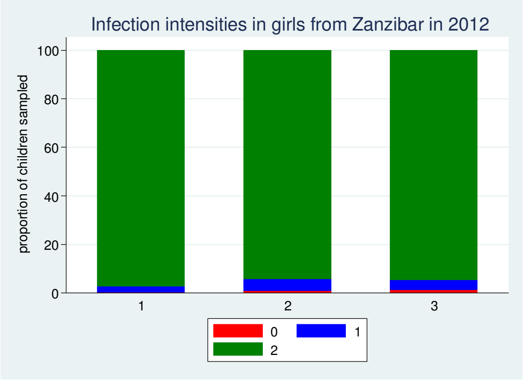

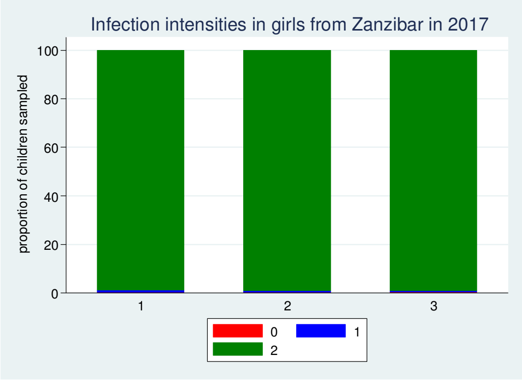

Boys

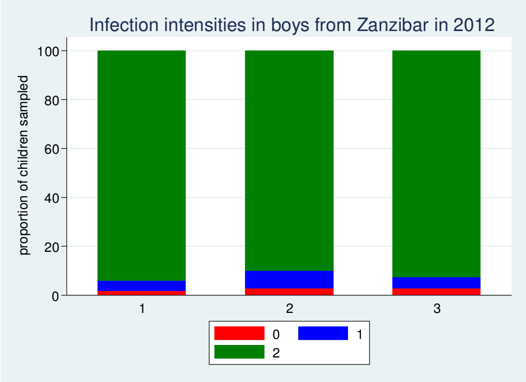

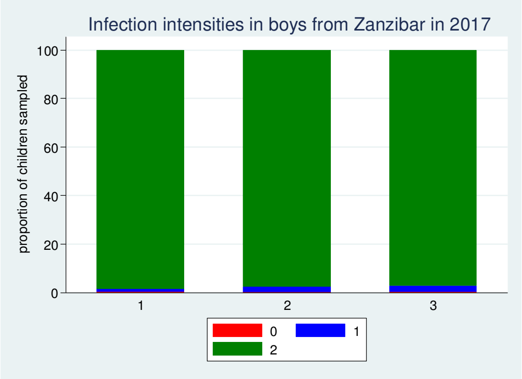

Figure 3b. Intensity categories by arm by year: Unguja (1=MDA Arm, 2=Snail Arm, 3=Behaviour Arm)

Overall

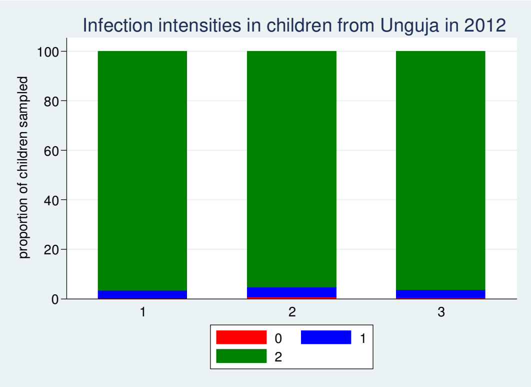

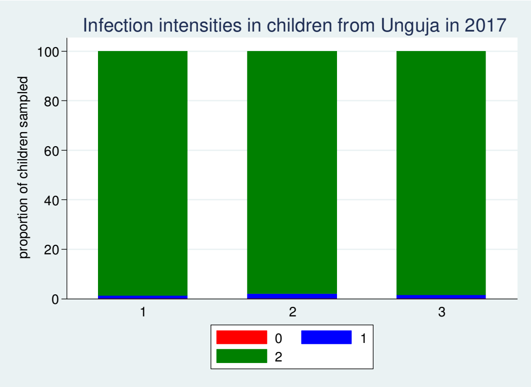

Girls

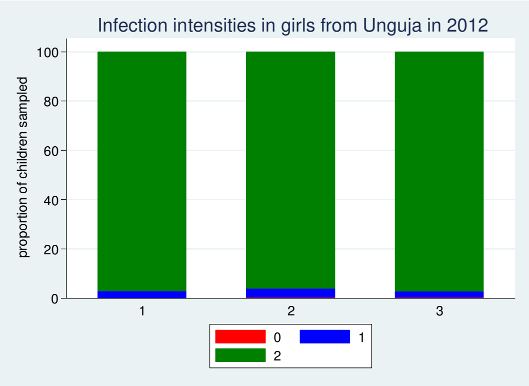

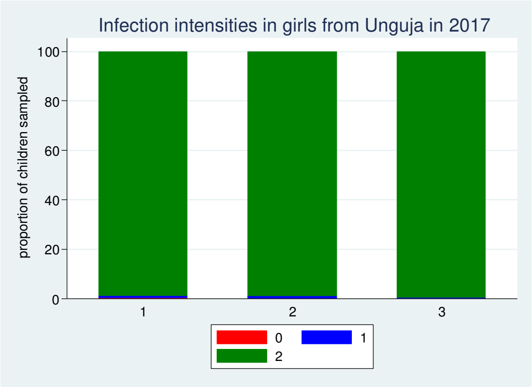

Boys

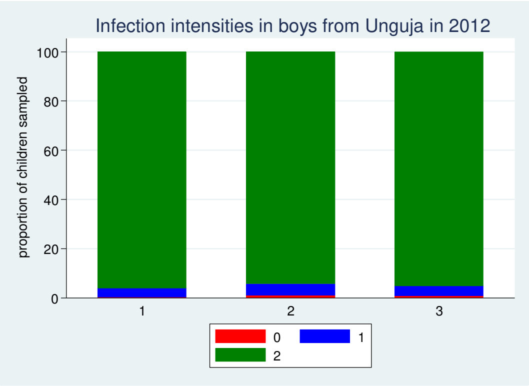

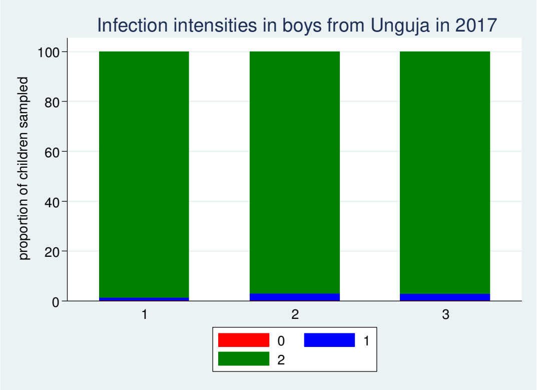

Figure 3c. Intensity categories by arm by year: Pemba (1=MDA Arm, 2=Snail Arm, 3=Behaviour Arm)

Overall

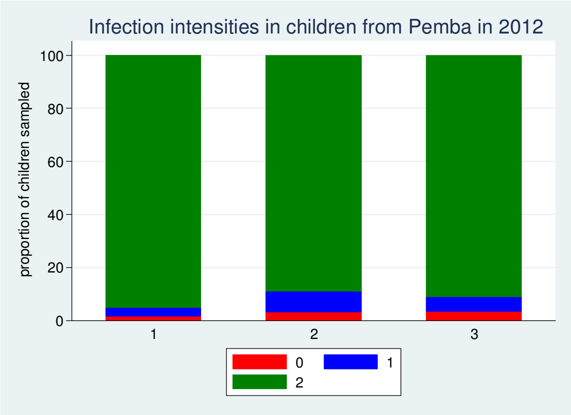

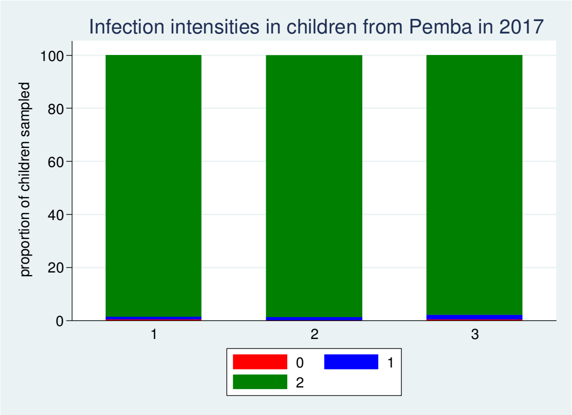

Girls

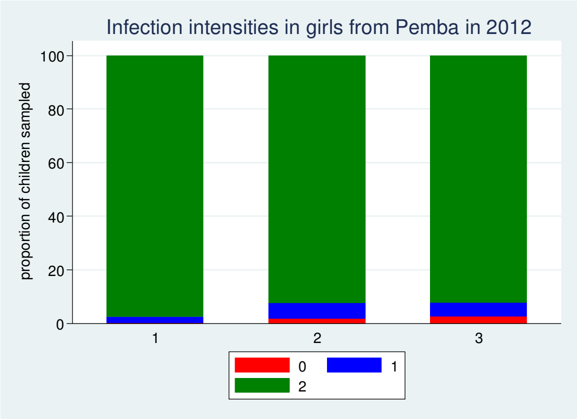

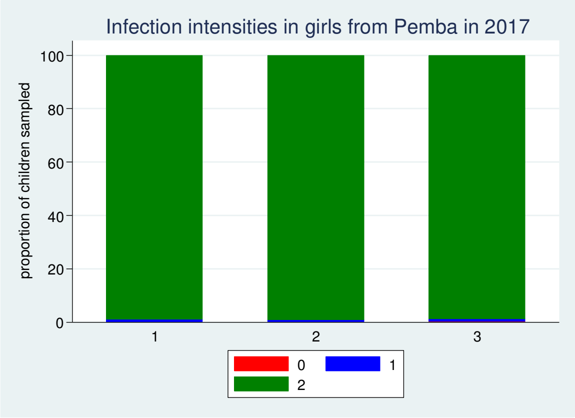

Boys

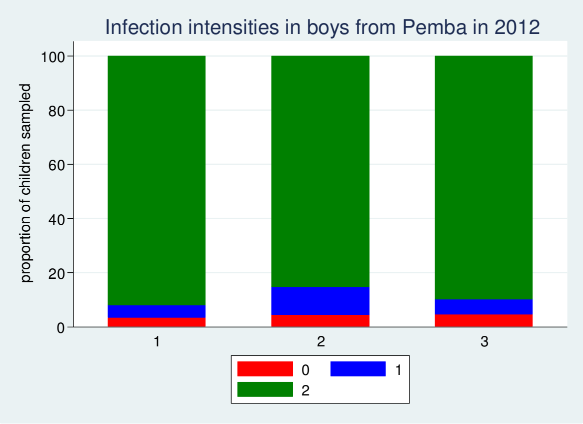

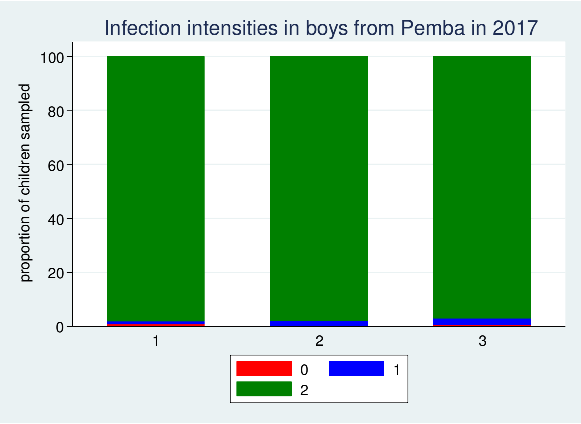

Supplemental table to Figure 3. Intensity categories by arm at baseline (2012) and in Year 6 (2017)

Island	Study Year	Study Arm	Infection Catergory	N	Study Year	Study Arm	Infection Catergory	N
Zanzibar	1	MDA	heavy	25	6	MDA	heavy	12
	1	MDA	light	93	6	MDA	light	35
	1	MDA	uninfected	2712	6	MDA	uninfected	3125
	1	Snail	heavy	49	6	Snail	heavy	8
	1	Snail	light	158	6	Snail	light	48
	1	Snail	uninfected	2451	6	Snail	uninfected	3157
	1	Behaviour	heavy	52	6	Behaviour	heavy	13
	1	Behaviour	light	111	6	Behaviour	light	45
	1	Behaviour	uninfected	2422	6	Behaviour	uninfected	3020
**Island**	**Study Year**	**Study Arm**	**Infection Catergory**	**N**	**Study Year**	**Study Arm**	**Infection Catergory**	**N**
Pemba	1	MDA	heavy	23	6	MDA	heavy	8
	1	MDA	light	47	6	MDA	light	17
	1	MDA	uninfected	1363	6	MDA	uninfected	1599
	1	Snail	heavy	39	6	Snail	heavy	4
	1	Snail	light	102	6	Snail	light	19
	1	Snail	uninfected	1133	6	Snail	uninfected	1583
	1	Behaviour	heavy	45	6	Behaviour	heavy	9
	1	Behaviour	light	70	6	Behaviour	light	25
	1	Behaviour	uninfected	1182	6	Behaviour	uninfected	1605
**Island**	**Study Year**	**Study Arm**	**Infection Catergory**	**N**	**Study Year**	**Study Arm**	**Infection Catergory**	**N**
Unguja	1	MDA	heavy	2	6	MDA	heavy	4
	1	MDA	light	46	6	MDA	light	17
	1	MDA	uninfected	1349	6	MDA	uninfected	1526
	1	Snail	heavy	10	6	Snail	heavy	4
	1	Snail	light	56	6	Snail	light	29
	1	Snail	uninfected	1318	6	Snail	uninfected	1574
	1	Behaviour	heavy	7	6	Behaviour	heavy	4
	1	Behaviour	light	41	6	Behaviour	light	20
	1	Behaviour	uninfected	1240	6	Behaviour	uninfected	1415

Infection intensity categories for *S. haematobium*: light 1-49 eggs/10 ml, heavy $\geq$50 eggs/10 ml.

Table 4a. Comparison of prevalence at Year 6 (2017) among arms, unadjusted

**Island**	**Unadjusted prevalence model estimate**		
**Zanzibar**	**OR**	**Lower 95% CI**	**Upper 95% CI**
Snail versus MDA	1.2	0.5	2.7
Behaviour versus MDA	1.3	0.6	2.9
Snail versus MDA - Female	0.8	0.3	2.7
Behaviour versus MDA - Female	0.8	0.2	2.7
Snail versus MDA - Male	1.5	0.7	3.2
Behaviour versus MDA - Male	1.8	0.9	3.5
Behaviour versus Snail	1.1	0.5	2.2
MDA versus Behaviour plus Snail	0.6	0.2	2.6
Snail versus MDA (weighted, age, sex logit model)	1.2	0.5	2.6
Behaviour versus MDA (weighted, age, sex logit model)	1.4	0.6	3.2
Behaviour versus Snail (weighted, age, sex logit model)	1.2	0.6	2.5
MDA versus Behaviour plus Snail (weighted, age, sex logit model)	0.6	0.1	2.4
Snail versus MDA (weighted, age, sex count model)	1.0	0.3	3.3
Behaviour versus MDA (weighted, age, sex count model)	1.8	0.6	6.0

OR: odds ratio

95% CI: 95 confidence intervals

Table 4b. Comparison of prevalence at Year 6 (2017) among arms, adjusted

Island	Adjusted prevalence model estimate		
**Zanzibar**	**OR**	**Lower 95% CI**	**Upper 95% CI**
Snail versus MDA	0.4	0.2	0.8
Behaviour versus MDA	0.4	0.2	0.8
Behaviour versus Snail	0.2	0.1	0.4
MDA versus Behaviour plus Snail	Non-est		

OR: odds ratio

95% CI: 95 confidence intervals

Table 4a. Comparison of intensity at Year 6 (2017) among arms, unadjusted and adjusted

Island	Unadjusted intensity ratio			Adjusted intensity ratio		
**Zanzibar**	**OR**	**Lower 95% CI**	**Upper 95% CI**	**OR**	**Lower 95% CI**	**Upper 95% CI**
Snail versus MDA	0.9	0.3	3.3	0.4	0.1	1.4
Behaviour versus MDA	1.4	0.4	4.4	0.5	0.1	1.8

OR: odds ratio

95% CI: 95 confidence intervals

Figure 4a. Mean intensity of *S. haematobium* by study arm over time: Zanzibar (both islands)

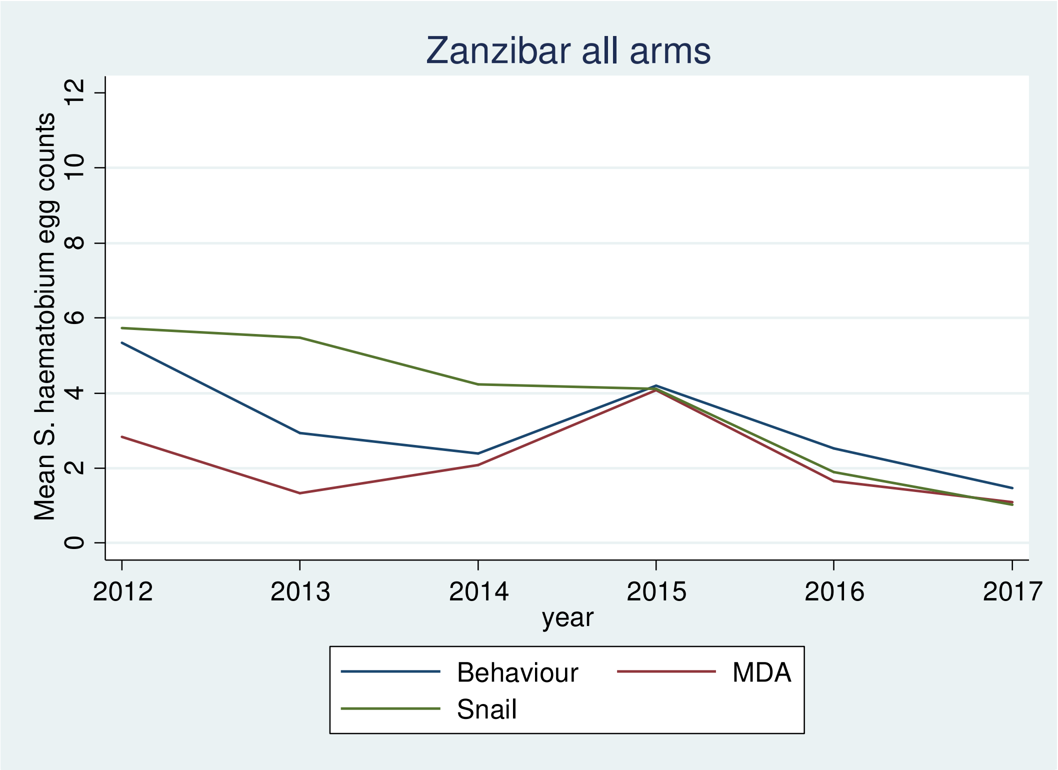

Figure 5b. Mean intensity of *S. haematobium* by study arm over time: Pemba

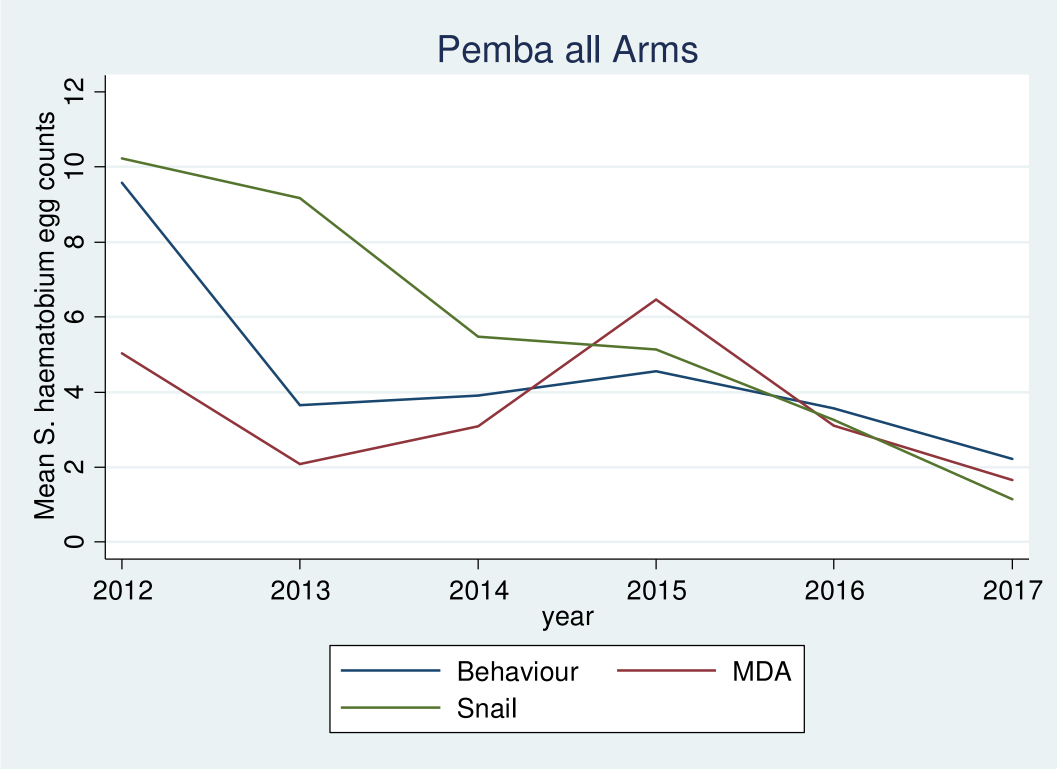

Figure 5c. Mean intensity of *S. haematobium* by study arm over time: Unguja

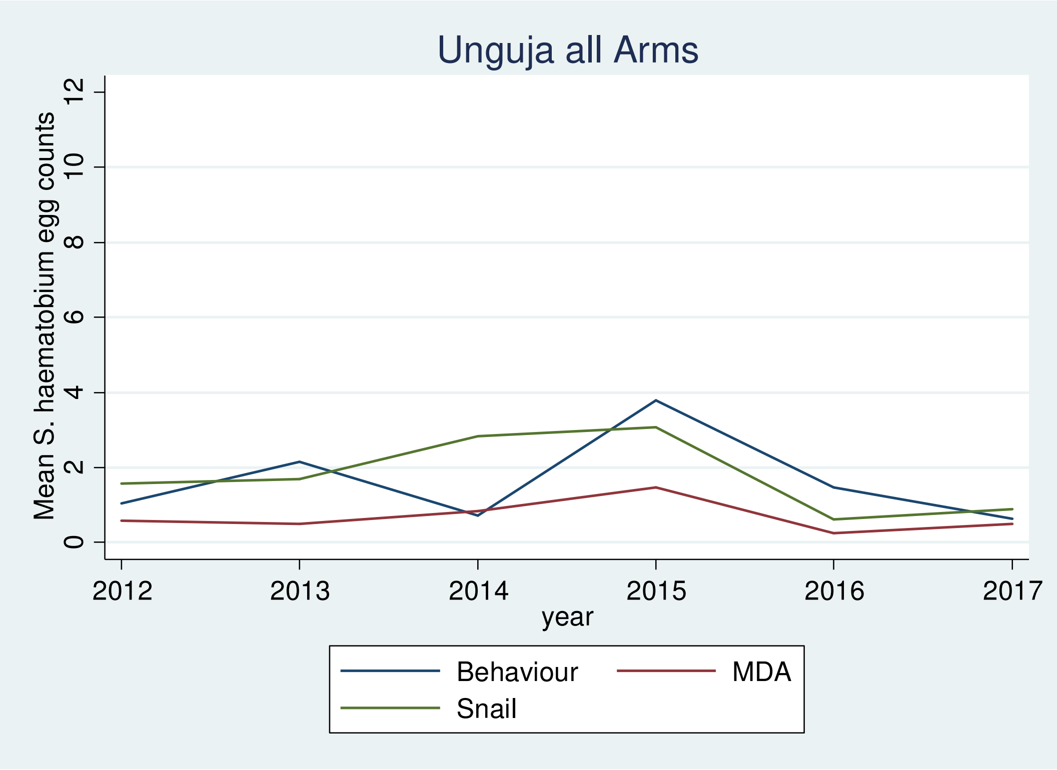

Figure 5a. Mean intensity of *S. haematobium* by school over time: Pemba

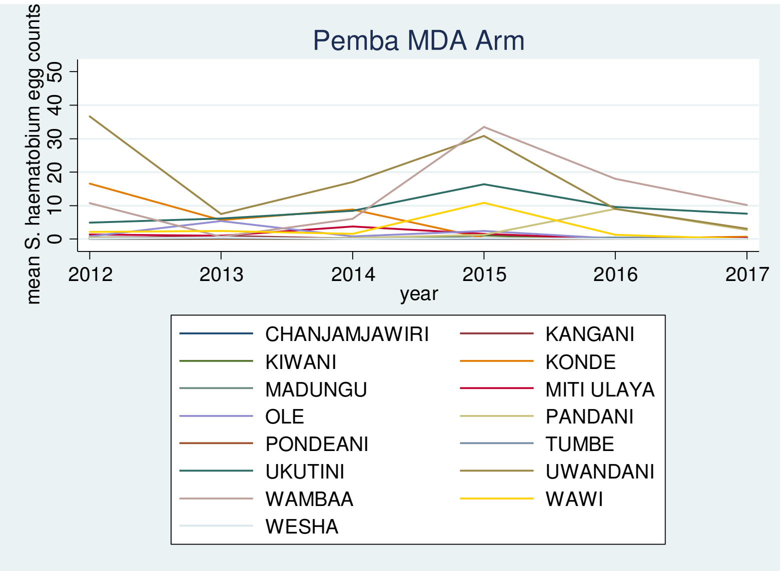

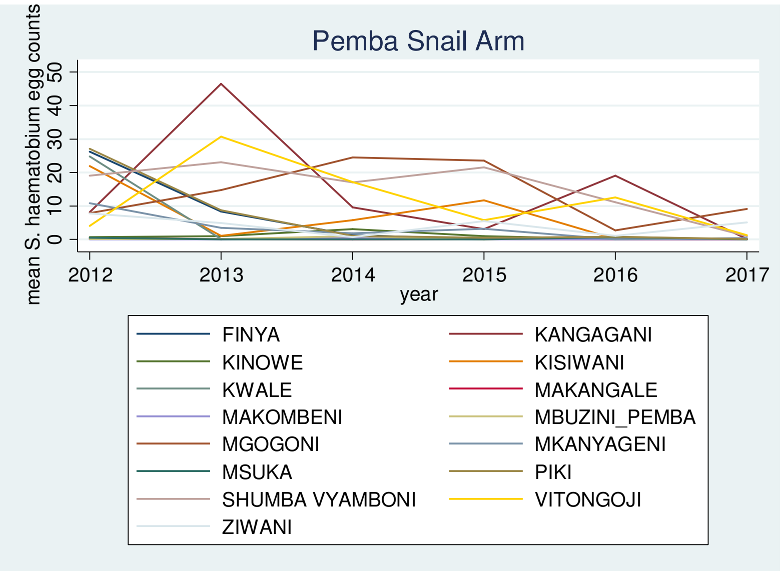

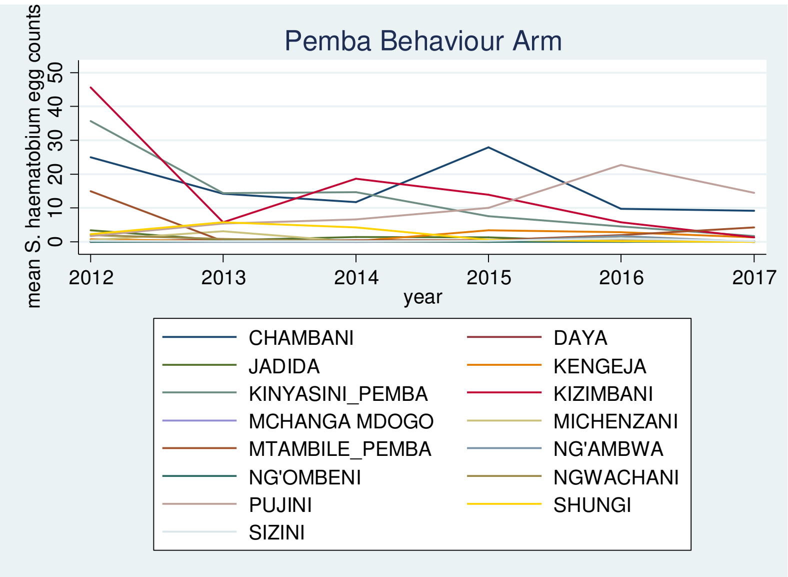

Figure 5b. Mean intensity of *S. haematobium* by school over time: Unguja

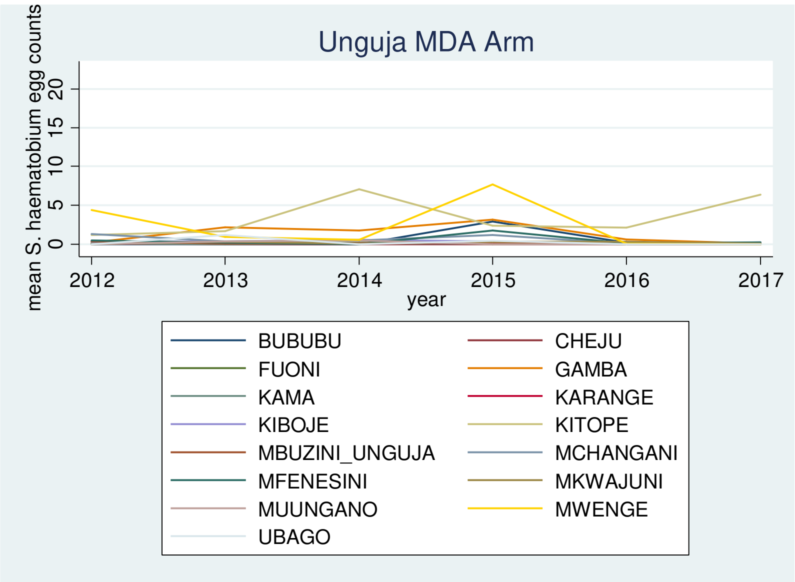

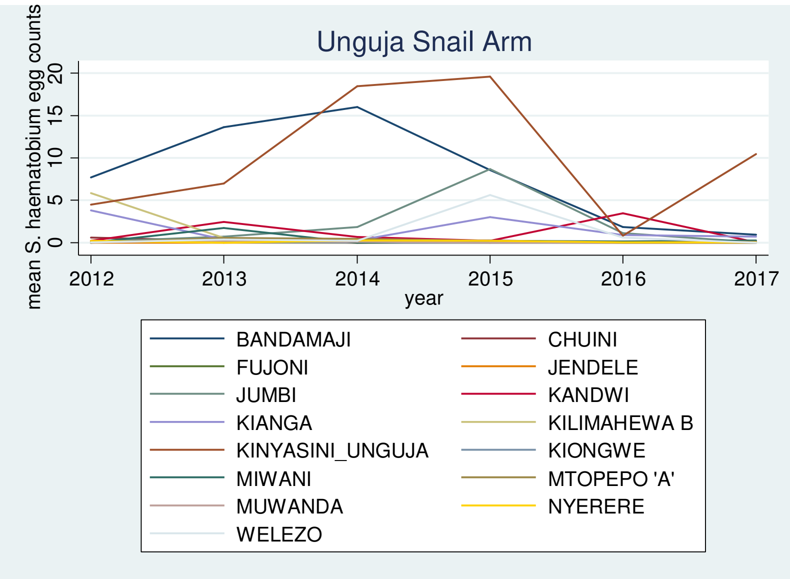

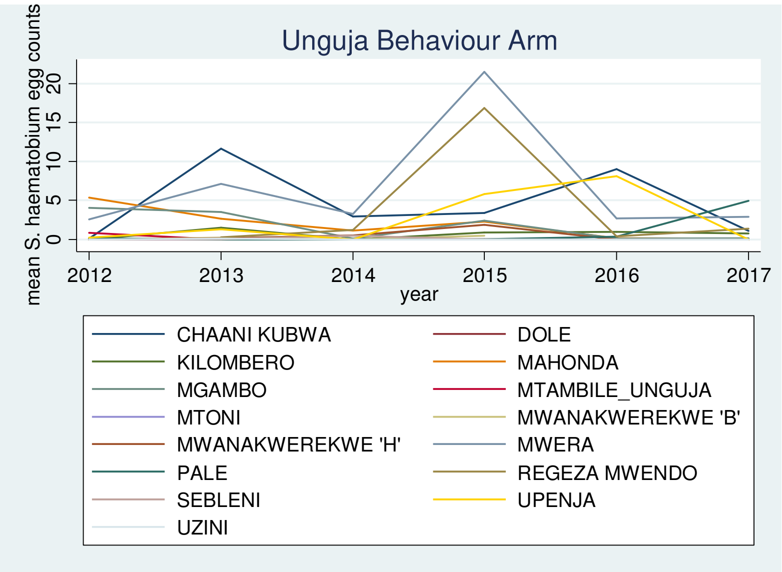

Supplement: Supplementary file 1 [file tpmd190825.SD1.pdf]
